# Evolution and Biological Evaluation of Matrinic Derivatives with Amantadine Fragments As New Anti-Influenza Virus Agents

**DOI:** 10.3390/molecules24050921

**Published:** 2019-03-06

**Authors:** Tianyu Niu, Xiaoqiang Zhao, Jing Jiang, Haiyan Yan, Yinghong Li, Sheng Tang, Yuhuan Li, Danqing Song

**Affiliations:** Beijing Key Laboratory of Anti-infective Agents, Institute of Medicinal Biotechnology, Chinese Academy of Medical Science & Peking Union Medical College, Beijing 10005, China; niutianyubasn@163.com (T.N.); xiaoqiangzhao@126.com (X.Z.); 18299093210@163.com(J.J.); yan0495@163.com(H.Y.); liyinghong@imb.pumc.edu.cn (Y.L.); songdanqingsdq@hotmail.com (D.S.)

**Keywords:** tricyclic matrinic derivatives, anti-virus, influenza A virus, H3N2, structure–activity relationship

## Abstract

A series of novel tricyclic matrinic derivatives with 11-adamantyl substitution were designed, synthesized, and evaluated for their activities against Influenza A H3N2 virus, based on the privileged structure strategy. Structure-activity relationship (SAR) analysis indicated that the introduction of an 11-adamantyl might be helpful for the potency. Among them, compounds **9f** and **9j** exhibited the promising anti-H3N2 activities with IC_50_ values of 7.2 μM and 10.2 μM, respectively, better than that of lead **1**. Their activities were further confirmed at the protein level. Moreover, compound **9f** displayed a high pharmacokinetic (PK) stability profile in whole blood and a safety profile in vivo. In primary mechanism, compound **9f** could inhibit the virus replication cycle at early stage by targeting M2 protein, consistent with that of the parent amantadine. This study provided powerful information for further strategic optimization to develop these compounds into a new class of anti-influenza agents.

## 1. Introduction

Influenza virus infection is a serious threat to the public health and economy with significant morbidity and mortality [1,2,3]. According to the World Health Organization, there are approximately 3 to 5 million severe cases caused by influenza viruses and death toll is up to 500,000 each year across the world [4]. Influenza viruses are often classified into three categories as Type A (IAV), Type B (IBV) and Type C (ICV) [5]. IAVs are the most common cause of human Influenza and responsible for several cases of pandemic influenza all over the world, such as “Spanish” influenza H1N1 virus pandemic in 1918 [6] and the influenza A H1N1 virus pandemic in 2009 [7] as well as “Hong Kong” H3N2 virus in 1968 [8]. The incidence of influenza A H3N2 has been higher than that of H1N1 since 2010, resulting in H3N2 becoming the most prevalent virus in the world. Though influenza vaccination is an effective strategy for controlling influenza infections [9], live attenuated and inactivated vaccines often show a decreased or no efficiency among the elderly [10], and they even fail to offer protection against hetero-subtype viruses [11]. Several drugs, including neuraminidaseinhibitors (oseltamivir, zanamivir) [12] and the matrix protein (M2) proton channel blockers (amantadine, rimantadine) [13], have been widely used to treat influenza virus infectionsin clinic. However, an increasing number of drug-resistant variants have appeared, especially oseltamivir-resistant strains [14,15], and therefore it is urgent that new therapeutic drugs are developed to combat the influenza virus. 

In our previous study, tricyclic matrinic compound 12*N*-*p*-carboxybenzyl matrinic butane (**1**), derived from Chinese natural alkaline matrine (**MT**), was first identified to have a moderate activity against influenza A virus H3N2 (IC_50_: 16.2 μM) with an unknown mode of action [16]. Its unique chemical scaffold and biological activity encouraged us to continue to conduct the new round of structure–activity relationship (SAR) study, aiming to better understand SAR of its kind and develop a novel class of anti-influenza agents, taking compound **1** as the lead.

**Figure 1:**
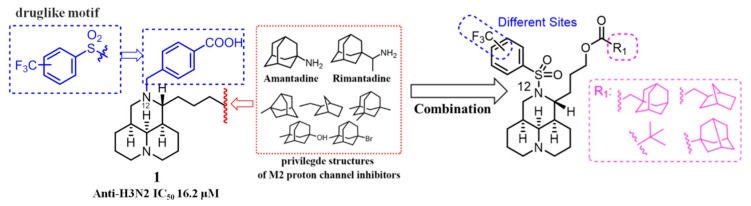
The strategy of structure modification.

According to our previous study, the replacement of the 12*N*-benzyl with 12*N*-trifluoromethylbenzensulfonyl motif led to a significant improvement in drug-like features [17], which shed light on our modification direction. In addition, the integration of an anti-influenza pharmacophore would be helpful. Rigid amantadine and rimantadine, as anti-influenza virus drugs, have been widely used in clinical practice for decades [13]. Therefore, based on the privileged segment combination strategy, besides the replacement of trifluoromethyl benzensulfonylmotif on the 12*N* atom, amantadine or rimantadine as a dominant fragment for activity was also introduced into 11-side chain terminus of tricyclic matrinic core, with which a series of hybrid compounds against influenza viruses were produced, as depicted in Figure 1. Therefore, in the present study, we described the synthesis of these novel matrinic derivatives with 11-rigid fragments, anti-influenza activities, and SAR analysis, as well as the stability profiles in whole blood, safety profiles in vivo, and the primary mechanism of action of key compounds.

## 2. Results and Discussion

### 2.1. Chemistry

**Scheme 1:**
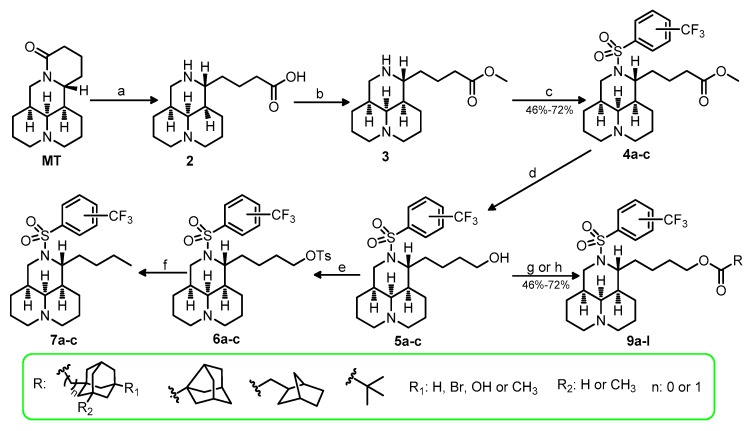
Reagents and conditions: (a) 5 N NaOH, reflux, 9 hr; 10 N HCl, pH = 3–5; (b) 2 N MeOH/HCl, reflux, 2 hr; (c) trifluoromethylbenzensulfonyl chloride, triethylamine (TEA), CH_2_Cl_2_, room temperature (r.t.), 6–8 hr; (d) LiAlH_4_, tetrahydrofuran (THF), 0 °C, 30 min; (e) TsCl, TEA, dimethylaminopyridine (DMAP), r.t., 6–8 h; (f) LiAlH_4_, THF, 0 °C to r.t., 30–60 min; (g) **8a**–**e**, SOCl_2_, r.t., 0.5 hr, TEA, r.t., 1 hr; (h) **8f**, CH_2_Cl_2_,TEA, r.t., 1 hr.

As described in Scheme 1, all of the title compounds were semi-synthesized with commercially available **MT** with purity over 98% as the starting material, which was purchased from the Yanchi Dushun Biological and Chemical Co. Ltd (Shanxi, China).

The key intermediates, methyl trifluoromethyl benzenesulfonyl matrinic butyrates (**4a**–**c**) were obtained by a three-step procedure, including hydrolysis, esterification, and 12*N*-sulfonylation from **MT**, with high yields of 70–75% [18]. Then, the reduction of the ester bond in the compounds **4a**–**c** by LiAlH_4_ achieved the intermediates 12*N*-trifluoromethyl benzenesulfonyl matrinic butanols (**5a**–**c**) respectively [19]. The target compounds 12*N*-trifluoromethyl benzenesulfonyl matrinic butanes (**7a**–**c**) were gained from **5a**–**c** by a two-step sequence reaction, including hydroxyl sulfonylation, and the reductive elimination of tosyloxy (OTs) by LiAlH_4_ at yields of 65–70% [20]. At the meantime, the hydroxyl esterification of the intermediates **5a** and 5**b** with the corresponding carboxylic acids or anhydrides (**8a**–**f**) gained another series of target compounds **9a**–**l** in yields of 46–72%. All of the title compounds were purified by flash column chromatography on silica gel using CH_2_Cl_2_/methanol as a gradient eluent.

### 2.2. Pharmacology

#### 2.2.1. SAR Analysis for Anti-H3N2 Activity in Vitro

The activity against influenza virus A/hanfang/359/95 (H3N2) of all target compounds was measured by the viral cytopathogenic effect (CPE) assay in Madin–Darby camine kidney (MDCK) cells with amantadine hydrochloride (**AH**) as the control drug. The activity against the H3N2 virus of each compound was evaluated by a combination of its IC_50_ and selectivity index (SI = TC_50_/IC_50_ ratio). The structures and the activities of all title compounds against H3N2 are shown in Table 1.

Firstly, the 11-*n*-butyl motif was retained, and the benzyl substitution on the 12-nitrogen atom was replaced with a more drug-like trifluoromethyl benzenesulfonyl substitution [17], by which three matrinane derivatives (**7a**–**c**) were synthesized and evaluated for their activities against H3N2. Compounds **7a**–**b** with *m*- or *p*-trifluoromethyl on benzene ring displayed higher activity than the *o*-trifluoromethyl counterpart **7c**. 

Therefore, the trifluoromethyl group on the *m*- or *p*- position of benzene ring was maintained, different rigid alkyl groups, including adamantyl, noradamantantyl, and norbornaneacetyl as privileged anti-influenza pharmacophores [13], were linked to the C11-side chain terminus by an ester bond, by which compounds **9a**–**l** were constructed. Among them, compounds **9a**–**f**, **9i**, and 9**j** bearing an adamantyl or a norbornaneacetyl group on the C11-butane chain terminus showed comparable or superior activities to lead **1**, while the noradamantantyl substituted compounds **9g** and 9**h** showed a complete loss in activity. Specifically, compounds **9f** with a 5-bromoadamantyl and **9j** with a norbornaneacetyl group were more potent than **1**, with IC_50_ values of 7.2 μM and 10.2 μM, respectively. At the meantime, it was worth noting that *m*-trifluoromethyl on the benzene ring might be more beneficial than the *p*- substitution, as compared between **9a**–**c** and **9d**–**f,** as well as **9i** and 9**j**, which was also speculated from the ranking result between **9k** and **9l**. Although the most potent compounds failed to display higher SI values than the lead **1** or **AH**, owing to their high cellular toxicities, this result provided a hint that the functionalization of C11-butane chain with bulky adamantyl or norbornaneacetyl ester groups might be beneficial for maintaining potency.

#### 2.2.2. Anti-H3N2 Activity of Key Compounds at the Protein Level

**Figure 2:**
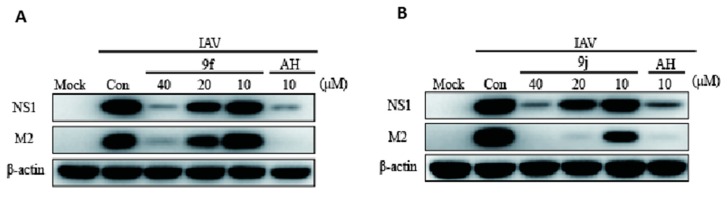
Compounds 9f and 9j exerted anti-H3N2 activities at the protein level. (**A** and **B**), MDCK cells were infected with IAV (H3N2, multiplicity of infection (MOI) = 0.003) in the presence or absence of compound 9f and 9j in different concentrations, and AH for 24 hr. The IAV NS1 and M2 expressions were analyzed by Western blot.

In order to further confirm the anti-influenza A virus activity of these compounds, the top compounds **9f** and **9j** with different structure types on the C11-side chain were selected to verify their effects against influenza virus H3N2 at protein level by Western Blot, taking **AH** as a positive reference. As indicated in Figure 2, both of them could significantly reduce H3N2 virus non-structural protein 1 (NS1, a homodimeric RNA-binding protein found in influenza virus required for viral replication [21]) level at a concentration of 40 μM, to a comparable extent to that of AH at the concentration of 10 μM, which is consistent with the effect at cell level. The results indicated that **9f** and **9j** were active against the influenza A H3N2 virus. 

#### 2.2.3. Anti-H1N1 Activity of Representative Compounds

Four top compounds **9c**, **9d**, **9f**, and **9j** with the highest anti-H3N2 activity and selectivity index were selected as the representative compounds to further evaluate their anti-influenza activities against influenza virus A/Fort Monmouth/1/1947(H1N1). As indicated in Table 1, all of the four target compounds also afforded potent activities against H1N1 virus, with an IC_50_ value of between 22 µM and 28 µM, much less than that of the control drug **AH** with IC_50_ value of 2.73 µM. 

#### 2.2.4. Safety and Metabolic Stability Assessments of Key Compounds

The acute toxicity tests of the two key compounds **9f** and **9j** were performed in Kunming mice. Each compound was given orally in a single-dosing experiment at 125, 250, and 500 mg·kg^–1^, respectively. Then, the mice were closely monitored for seven days. Both of them had good safety profiles with median lethal dose (LD_50_) values higher than 500 mg·kg^–1^, and neither significant weight loss nor behavioral abnormality was noted in all treatment groups as compared to the control (data not shown), indicating a satisfactory safety profile in vivo. 

**Figure 3:**
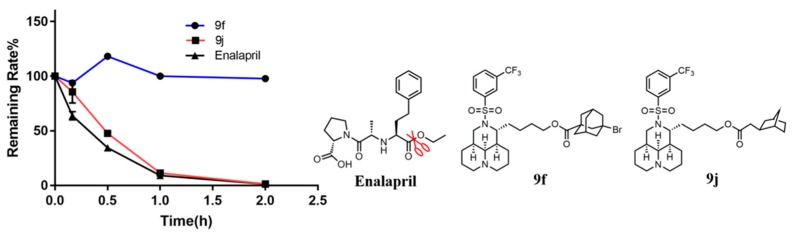
The stability assessments of key compounds **9f** and **9j** in whole blood. The dosing solutions were taken at 0, 1, 5, 10, 20, 40, 60, and 120 min, respectively, after the incubation of blood and tested samples at 37 °C in a water bath.

Owing to the existence of hydrolyzable ester bond, the stability of compounds **9f** and **9j** in whole blood in vitro was investigated, taking prodrug (*S*)-*N*-[1-(ethoxycarbonyl)-3-phenylpropyl]-Ala-Pro maleate (enalapril) as a positive reference, which also had an ester bond in its structure and was thus easy to be hydrolyzed by esterase. As shown in Figure 3, enalapril was hydrolyzed quickly in blood, as expected. Compound **9j** displayed a poor stability in blood, and its remaining rates of protype were 47.7% at 30 min, and 1.3% at 120 min, respectively, similar to the positive reference, enalapril. However, compound **9f** had a high stability profile, and its remaining rates were 100% at 60 min, and 97.9% at 120 min, respectively, resulting from the higher steric hindrance of the adamantyl to protect the ester bond from hydrolyzing by esterase. The results hinted that **9f** owned a good PK stability profile in blood, and it was chosen for the next investigation.

#### 2.2.5. Primary Mechanism of **9f**

Therefore, compound **9f** was selected as a representative one for the primary mechanisms of action study against influenza. As amantadine and rimantadine displayed anti-influenza efficacy by targeting the M2 proton channel, the inhibition of **9f** for M2 was initially tested by Western blot. As shown in Figure 2, the compound **9f** could significantly reduce the M2 level at the concentration of 40 μM, consistent with that of the parent **AH** at a concentration of 10 μM.

**Figure 4:**
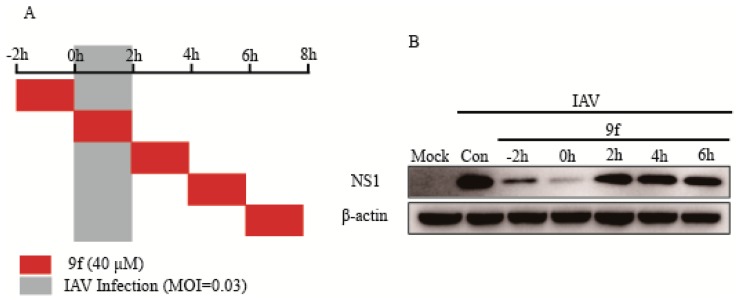
Compound **9f** inhibited the influenza virus replication cycle at an early stage according to the time-of-addition study. (**A**) Schematic of the experiment to determine which stage of the viral cycle is inhibited by compound **9f** in MDCK cells. (**B**) MDCK cells infected with IAV (H3N2, MOI = 0.03) were treated with compound **9f** (40μM) either at −2 h, 0, 2, 4 or 6 h p.i., and virus NS1 expression were detected by Western blot at 8 hp.i.

In order to further confirm the mode of action of this kind of compound against the influenza virus, a time-of-addition experiment for **9f** was carried out as well. The therapeutic efficacy of **9f** at different infection time points (before, at, and after infection) was measured respectively, and the results are shown in Figure 4. It demonstrated that compound **9f** was effective at −2 hr and 0 hr of H3N2 infection, and no significant changes were observed in the later periods, indicating that **9f** might inhibit the virus replication cycle at an early stage, consistent with the mechanism of inhibition of the M2 protein.

## 3. Experimental Section

### 3.1. Chemistry

Unless otherwise noted, all chemical reagents were obtained from commercial sources and used without further purification. Melting points (m.p.) were obtained with the CXM-300 melting point apparatus. ^1^H NMR and ^13^C NMR spectra were recorded on Bruker Avanced spectrometer (Varian, San Francisco, CA, USA) (600 MHz, 500 MHz and 400 MHz for ^1^H NMR; 151 MHz, 126 MHz, and 101 MHz for ^13^C NMR) in DMSO-*d*_6_. ESI high-resolution mass spectra (HRMS) were recorded on an Autospec Ultima-TOF spectrometer (Micromass UK Ltd., Manchester, UK). The normalization method of HPLC analysis was as follows: column, Inertsustain C18, 250 mm×4.6 mm, id; flow rate, 1.0 mL/min; mobile phase, acetonitrile/water (0.01 mol·L^−1^ KH_2_PO_4_ in water, pH = 4.0) = 5:95; Flash column chromatography was performed on Combiflash Rf 200 (Teledyne, Lincoln, NE, USA), particle size: 0.038 mm.

#### 3.1.1. General Procedure for the Synthesis of Compounds **5a–c**

**MT** (10.0 g, 40 mmol) was added to 5 N NaOH solution (50 mL). The mixture was refluxed for 9 hr and cooled overnight to a precipitate, and many solids were precipitated. The solid was then transferred into concentrated HCl (10 N), and the pH of the solution was maintained at less than 4. The solvent was removed in vacuo, and the residue was dissolved with methanol (50 mL). Filtered the suspension, and the filtrate was concentrated. The residue was dissolved in 2 N HCl/MeOH (50 mL) and the mixture was refluxed for 2 hr. The solvent was removed under reduced pressure to give a crude product, which was purified by recrystallization from ethanol to achieve **3** (13.1 g). 

To a suspension of compound **3** (10.0 g, 28 mmol) in CH_2_Cl_2_ (50 mL), TEA (8.68 g, 86 mmol) and *p*-trifluoromethylbenzenesulfonyl chloride (34 mmol) were added, the reaction mixture was stirred for 6 hr at r.t. until thin-layer chromatography (TLC) analysis showed that the reaction was complete. The reaction mixture was then washed by saturated aqueous ammonium chloride (50 mL × 2), and saturated aqueous sodium chloride (50 mL), subsequently dried over anhydrous Na_2_SO_4_, and concentrated under reduced pressure. The residue was purified by recrystallization from methanol to give **4a**.

To a solution of **4a** (10 mmol), anhydrous THF (50 mL) and LiAlH_4_ (11 mmol) were added in an ice bath, and the mixture was stirred for 0.5 hr before being quenched by methanol and saturated aqueous ammonium chloride (2.5 mL). This was poured into CH_2_Cl_2_ (200 mL) and filter through diatomite. The solution was concentrated under reduced pressure to obtained the crude product **5a**, which was used directly without further isolation. Similarly, compounds **5b** and **5c** were obtained from **3**, and the corresponding trifluoromethyl benzenesulfonyl chloride in the same manner as **5a**. Compounds **7a**–**c** have been previously reported [17].

#### 3.1.2. General Procedure for the Synthesis of Compounds **9a–j**

**Figure 5:**
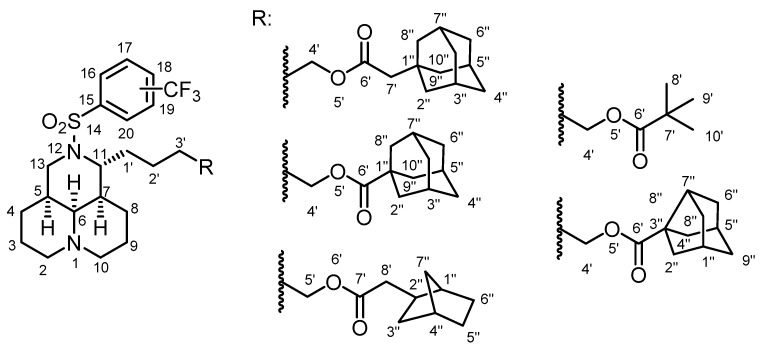
structures and position numbers of the target compounds **9a**–**j**.

**8a**–**e** (1.0 mmol) were dissolved in thionyl chloride (5 mL), and the mixture was stirred for 1 hr at r.t. Then, the solvent was removed by condensation, the residue was dissolved in CH_2_Cl_2_ (10 mL), and it was added into a solution of **5a** or **5b** (1.1 mmol) and TEA (1.5 mmol) in CH_2_Cl_2_ (30 mL), and the solution was stirred at r.t. until TLC analysis showed that the reaction was complete. The solution was washed by saturated aqueous ammonium chloride (50 mL × 2), saturated aqueous sodium chloride (50 mL), dried over anhydrous Na_2_SO_4_, and concentrated under reduced pressure. The residue was purified by flash column chromatography on a silica gel with CH_2_Cl_2_/CH_3_OH as the eluent, and treated with 2 N HCl/ether (3 mL) to yield the desired products. Their structures and position numbers are shown in Figure 5. ^1^H NMR and ^13^CNMR spectrum of compounds **9a–9j** see Supplementary Materials.

*12N-p-Trifluoromethlybenzenesulfonyl matrinic butyl adamantine-1’-carboxylate hydrochloride* (**9a**). Compound **5a** (0.50 g, 1.1 mmol) was treated with 1-adamantanecarboxylic acid (**8a**, 0.18 g, 1.0 mmol) according to the general procedure, to yield the product **9a** as a white solid, yield: 69%; m.p.: 231 °C (dec.); ^1^H NMR (600 MHz): 10.68 (s, 1H, 1-NH^+^), 8.07 (d, *J* = 8.1 Hz, 2H, 16-CH, 20-CH), 7.98 (d, *J* = 8.1 Hz, 2H, 17-CH, 19-CH), 4.17–4.11 (m, 1H, 11-CH), 3.88–3.69 (m, 4H, 13-CH_2_, 4’-CH_2_), 3.59–3.55 (m, 1H, 6-CH), 3.19 (d, *J* = 11.7 Hz, 2H, 2-CH_2_), 2.93–2.84 (m, 2H, 10-CH_2_), 2.31–2.24 (m, 2H, 5-CH, 7-CH), 1.96–1.83 (m, 5H, 3’’-CH, 5’’-CH, 7’’-CH, 8-CH_2_), 1.75–1.73 (m, 6H, 2’’-CH_2_, 8’’-CH_2_, 9’’-CH_2_), 1.70–1.54 (m, 14H, 4-CH_2_, 9-CH_2_,1’-CH_2_, 2’-CH_2_, 4’’-CH_2_, 6’’-CH_2_, 10’’-CH_2_), 1.46–1.40 (m, 1H, 3’a-CH), 1.31–1.23 (m, 1H, 3’b-CH), 1.20–1.12 (m, 1H, 3a-CH), 0.97–0.88 (m, 1H, 3b-CH); ^13^C NMR (151 MHz) δ 176.3 (6’-C), 145.2 (15-C), 132.4 (18-C), 127.7 (17-C, 19-C), 126.6 (16-C, 20-C), 126.4 (21-C), 63.1 (6-C), 62.9 (4’-C), 57.8 (11-C), 54.7 (10-C), 54.5 (2-C), 48.5 (13-C), 38.4 (1’’-C), 38.3 (2’’-C, 8’’-C, 9’’-C), 38.1 (7-C), 35.9 (4’’-C, 6’’-C, 10’’-C), 34.6 (5-C), 27.8 (3’-C), 27.4 (1’-C), 27.3 (3’’-C, 5’’-C, 7’’-C), 24.8 (4-C), 24.3 (8-C), 21.7 (3-C), 18.3 (9-C), 18.1 (2’-C); HRMS *m/z*:Calcd for C_33_H_46_O_4_N_2_F_3_S [M–HCl+H]^+^, 623.3125; found, 623.3115; HPLC: *t_R_* =19.25 min, purity 95.58%.

*12N-p-Trifluoromethlybenzenesulfonyl matrinic butyl-1’-adamantaneacetatehydrochloride* (**9b**). Compound **5a** (0.50 g, 1.1 mmol) was treated with 1-adamantaneacetic acid (**8b**, 0.19 g, 1.0 mmol) according to the general procedure to yieldthe product 9**b** as a white solid, yield: 63%; m.p.: 227 °C (dec.); ^1^H NMR (600 MHz) δ 10.71–10.59 (m, 1H, 1-NH^+^), 8.07 (d, *J* = 8.2 Hz, 2H, 16-CH, 20-CH), 7.98 (d, *J* = 8.2 Hz, 2H, 17-CH, 19-CH), 4.16–4.09 (m, 1H, 11-CH), 3.83 (t, *J* = 13.0 Hz, 1H, 13a-CH), 3.79–3.75 (m, 1H, 13b-CH), 3.73 (t, *J* = 6.6 Hz, 2H, 4’-CH_2_), 3.60–3.54 (m, 1H, 6-CH), 3.19 (d, *J* = 11.9 Hz, 2H, 2-CH_2_), 2.94–2.85 (m, 2H, 10-CH_2_), 2.29–2.22 (m, 2H, 5-CH, 7-CH), 1.96 (s, 2H, 7’-CH_2_), 1.93–1.86 (m, 5H, 3’’-CH, 5’’-CH, 7’’-CH, 8-CH_2_), 1.74–1.61 (m, 10H, 2’’-CH_2_, 8’’-CH_2_, 9’’-CH_2_, 4-CH_2_, 1’-CH_2_), 1.57–1.49 (m, 10H, 9-CH_2_, 2’-CH_2,_ 4’’-CH_2_, 6’’-CH_2_, 10’’-CH_2_), 1.49–1.39 (m, 1H, 3’a-CH), 1.31–1.20 (m, 1H, 3’b-CH), 1.20–1.10 (m, 1H, 3a-CH), 0.95–0.85 (m, 1H, 3b-CH); ^13^C NMR (151 MHz) δ 170.6 (6’-C), 145.2 (15-C), 132.4 (18-C), 127.7 (17-C, 19-C), 126.6 (16-C, 20-C), 123.4 (21-C), 63.1 (6-C), 62.8 (4’-C), 57.8 (11-C), 54.6 (10-C), 54.5 (2-C), 48.5 (13-C), 48.1 (7’-C), 41.7 (2’’-C, 8’’-C, 9’’-C), 38.1 (7-C), 36.2 (4’’-C, 6’’-C, 10’’-C), 34.6 (5-C), 32.1 (1’’-C), 27.9 (3’’-C, 5’’-C, 7’’-C), 27.9 (3’-C), 27.5 (1’-C), 24.8 (4-C), 24.3 (8-C), 22.0 (3-C), 18.3 (9-C), 18.1 (2’-C); HRMS *m/z*:Calcd for C_34_H_48_O_4_N_2_F_3_S [M–HCl+H]^+^, 637.3281; found, 637.3272; HPLC: *t_R_* = 19.14 min, purity 99.28%.

*12N-p-Trifluoromethlybenzenesulfonylmatrinicbutyl-3’-bromoadamantane-1’-carboxylate hydrochloride* (**9c**). Compound **5a** (0.50 g, 1.1 mmol) was treated with 3-bromo-1-adamantanecarboxylic acid (**8c**, 0.26 g, 1.0 mmol) according to the general procedure to yield the product **9c** as a white solid, yield: 86%; m.p.: 222–223 °C; ^1^H NMR (600 MHz) δ 10.68 (d, *J* = 9.8 Hz, 1H, 1-NH^+^), 8.07 (d, *J* = 8.2 Hz, 2H, 16-CH, 20-CH), 7.98 (d, *J* = 8.2 Hz, 2H, 17-CH, 19-CH), 4.13 (dt, *J* = 11.5, 5.9 Hz, 1H, 11-CH), 3.81–3.74 (m, 4H, 4’-CH_2_, 13-CH_2_), 3.60–3.53 (m, 1H, 6-CH), 3.24–3.13 (m, 2H, 2-CH_2_), 2.93–2.84 (m, 2H, 10-CH_2_), 2.34 (s, 2H, 2’’-CH_2_), 2.29–2.22 (m, 6H, 5-CH, 7-CH, 5’’-CH, 7’’-CH, 4’’a-CH, 10’’a-CH), 2.14–2.09 (m, 2H, 4’’b-CH, 10’’b-CH), 1.92–1.84 (m, 2H, 8-CH_2_), 1.77–1.74 (m, 4H, 8’’-CH_2_, 9’’-CH_2_), 1.70–1.56 (m, 10H, 1’-CH_2_, 4-CH_2_, 9-CH_2_, 2’-CH_2_, 6’’-CH_2_), 1.49–1.41 (m, 1H, 3’a-CH), 1.33–1.25 (m, 1H, 3’b-CH), 1.21–1.12 (m, 1H, 3a-CH), 0.96–0.87 (m, 1H, 3b-CH); ^13^C NMR (151 MHz) δ 174.4 (6’-C), 145.2 (15-C), 132.5 (18-C), 127.7 (17-C, 19-C), 126.6 (16-C, 20-C), 123.5 (21-C), 66.0 (3’’-C), 63.5 (6-C), 62.9 (4’-C), 57.8 (11-C), 54.6 (10-C), 54.5 (2-C), 49.3 (13-C), 48.5 (2’’-C), 47.6 (8’’-C, 9’’-C), 44.5 (6’’-C), 38.1 (7-C), 36.5 (4’’-C, 10’’-C), 34.6 (5-C), 33.7 (1’’-C), 31.3 (5’’-C, 7’’-C), 27.8 (3’-C), 27.4 (1’-C), 24.8 (4-C), 24.4 (8-C), 21.9 (3-C), 18.3 (9-C), 18.1 (2’-C); HRMS *m/z*: Calcd for C_33_H_45_O_4_N_2_BrF_3_S [M–HCl+H]^+^, 701.2230; Found, 701.2230; HPLC: *t_R_* = 19.56 min, purity 95.58%.

*12N-m*-*Trifluoromethlybenzenesulfonyl matrinic butyl adamantine-1’-carboxylate hydrochloride* (**9d**). Compound **5b** (0.50 g, 1.1 mmol) was treated with 1-adamantanecarboxylic acid (**8a**, 0.18 g, 1.0 mmol) according to the general procedure to yield product **9d** as a white solid, yield: 38%; m.p.: 190–191 °C; ^1^H NMR (600 MHz) δ 10.83–10.75 (m, 1H, 1-NH^+^), 8.18 (d, *J* = 7.9 Hz, 1H, 18-CH), 8.07 (d, *J* = 7.9 Hz, 1H, 20-CH), 8.04 (s, 1H, 16-CH), 7.88 (t, *J* = 7.9 Hz, 1H, 19-CH), 4.23–4.18 (m, 1H, 11-CH), 3.89–3.78 (m, 2H, 13-CH_2_), 3.76–3.68 (m, 2H, 4’-CH_2_), 3.61–3.57 (m, 1H, 6-CH), 3.21–3.16 (m, 2H, 2-CH_2_), 2.93–2.85 (m, 2H, 10-CH_2_), 2.31–2.24 (m, 2H, 5-CH, 7-CH), 1.95–1.84 (m, 5H, 3’’-CH, 5’’-CH, 7’’-CH, 8-CH_2_), 1.75–1.73 (m, 6H, 2’’-CH_2_, 8’’-CH_2_, 9’’-CH_2_), 1.71–1.54 (m, 14H, 4-CH_2_, 9-CH_2_, 1’-CH_2_, 2’-CH_2_, 4’’-CH_2_, 6’’-CH_2_, 10’’-CH_2_), 1.47–1.36 (m, 1H, 3’a-CH), 1.28–1.20 (m, 1H, 3’b-CH), 1.12–1.03 (m, 1H, 3a-CH), 0.90–0.79 (m, 1H, 3b-CH); ^13^C NMR (151 MHz) δ 176.2 (6’-C), 143.0 (15-C), 131.2 (17-C), 130.8 (20-C), 129.8 (19-C), 129.5 (18-C), 123.6 (21-C), 122.9 (16-C), 63.1 (6-C), 63.0 (4’-C), 58.1 (11-C), 54.6 (10-C), 54.5 (2-C), 48.7 (13-C), 38.4 (1’’-C), 38.3 (2’’-C, 8’’-C, 9’’-C), 38.2 (7-C), 35.9 (4’’-C, 6’’-C, 10’’-C), 34.8 (5-C), 27.7 (3’-C), 27.4 (3’’-C, 5’’-C, 7’’-C), 27.3 (1’-C), 24.8 (4-C), 24.4 (8-C), 22.3 (3-C), 18.3 (9-C), 18.1 (2’-C); HRMS *m/z*:Calcd for C_33_H_46_O_4_N_2_F_3_S [M–HCl+H]^+^, 623.3125; found, 623.3115; HPLC: *t_R_* =18.89 min, purity 98.03%.

*12N*-*m*-*Trifluoromethlybenzenesulfonyl matrinic butyl-1*’*-adamantaneacetatehydrochloride* (**9e**). Compound **5b** (0.50 g, 1.1 mmol) was treated with 1-adamantaneacetic acid (**8b**, 0.19 g, 1.0 mmol) according to the general procedure to yieldproduct **9e** as a white solid, yield: 41%; m.p.: 155–156 °C; ^1^H NMR (600 MHz) δ 10.68 (s, 1H, 1-NH^+^), 8.18 (d, *J* = 8.0 Hz, 1H, 18-CH), 8.07 (d, *J* = 8.0 Hz, 1H, 20-CH), 8.04 (s, 1H, 16-CH), 7.88 (t, *J* = 8.0 Hz, 1H, 19-CH), 4.21–4.14 (m, 1H, 11-CH), 3.87–3.77 (m, 2H, 13-CH_2_), 3.71 (t, *J* = 6.6 Hz, 2H, 4’-CH_2_), 3.60–3.56 (m, 1H, 6-CH), 3.19 (d, *J* = 11.8 Hz, 2H, 2-CH_2_), 2.93–2.84 (m, 2H, 10-CH_2_), 2.30–2.24 (m, 2H, 5-CH, 7-CH), 1.95 (s, 2H, 7’-CH_2_), 1.92–1.85 (m, 5H, 3’’-CH, 5’’-CH, 7’’-CH, 8-CH_2_), 1.71–1.62 (m, 9H, 2’’-CH_2_, 8’’-CH_2_, 9’’-CH_2_, 4a-CH, 1’-CH_2_), 1.59–1.54 (m, 5H, 4b-CH, 9-CH_2_, 2’-CH_2_), 1.51 (d, *J* = 2.8 Hz, 6H, 4’’-CH_2_, 6’’-CH_2_, 10’’-CH_2_), 1.46–1.38 (m, 1H, 3’a-CH), 1.27–1.17 (m, 1H, 3’b-CH), 1.14–1.04 (m, 1H, 3a-CH), 0.89–0.80 (m, 1H, 3b-CH); ^13^C NMR (151 MHz) δ 170.6 (6’-C), 143.0 (15-C), 131.1 (17-C), 130.8 (20-C), 129.9 (19-C), 129.5 (18-C), 123.6 (21-C), 122.9 (16-C), 63.0 (6-C), 62.9 (4’-C), 58.1 (11-C), 54.6 (10-C), 54.5 (2-C), 48.5 (13-C), 48.1 (7’-C), 41.7 (2’’-C, 8’’-C, 9’’-C), 38.2 (7-C), 36.2 (4’’-C, 6’’-C, 10’’-C), 34.8 (5-C), 32.1 (1’’-C), 27.9 (3’’-C, 5’’-C, 7’’-C), 27.8 (3’-C), 27.4 (1’-C), 24.8 (4-C), 24.4 (8-C), 22.3 (3-C), 18.3 (9-C), 18.1 (2’-CH_2_); HRMS *m/z*:Calcd for C_34_H_48_O_4_N_2_F_3_S [M–HCl+H]^+^, 637.3281; found, 637.3265; HPLC: *t_R_*=19.63 min, purity 98.52%.

*12N-m-Trifluoromethlybenzenesulfonylmatrinicbutyl-3’-bromoadamantane-1’-carboxylate hydrochloride* (**9f**). Compound **5b** (0.50 g, 1.1 mmol) was treated with 3-bromo-1-adamantanecarboxylic acid (**8c**, 0.26 g, 1.0 mmol) according to the general procedure to yield product **9f** as a white solid, yield: 78%; m.p.: 221–222 °C; ^1^H NMR (600 MHz) δ 10.56 (s, 1H, 1-NH^+^), 8.18 (d, *J* = 7.8 Hz, 1H, 18-CH), 8.08 (d, *J* = 7.8 Hz, 1H, 20-CH), 8.05 (s, 1H, 16-CH), 7.88 (t, *J* = 7.8 Hz, 1H, 19-CH), 4.17–4.12 (m, 1H, 11-CH), 3.82–3.74 (m, 4H, 4’-CH_2_, 13-CH_2_), 3.57 (dt, *J* = 10.5, 3.4 Hz, 1H, 6-CH), 3.20 (d, *J* = 11.8 Hz, 2H, 2-CH_2_), 2.93–2.84 (m, 2H, 10-CH_2_), 2.34 (s, 2H, 2’’-CH_2_), 2.30–2.21 (m, 6H, 5-CH, 7-CH, 5’’-CH, 7’’-CH, 4’’b-CH, 10’’a-CH), 2.15–2.10 (m, 2H, 4’’b-CH, 10’’b-CH), 1.92–1.82 (m, 2H, 8-CH_2_), 1.76–1.74 (m, 4H, 8’’-CH_2_, 9’’-CH_2_), 1.71–1.55 (m, 10H, 1’-CH_2_, 4-CH_2_, 9-CH_2_, 2’-CH_2_, 6’’-CH_2_), 1.47–1.38 (m, 1H, 3’a-CH), 1.30–1.22 (m, 1H, 3’b-CH), 1.12–1.05 (m, 1H, 3a-CH), 0.91–0.83 (m, 1H, 3b-CH); ^13^C NMR (151 MHz) δ174.4 (6’-C), 142.9 (15-C), 131.2 (17-C), 130.8 (20-C), 129.9 (19-C), 129.5 (18-C), 123.4 (21-C), 122.9 (16-C), 66.0 (3’’-C), 63.5 (6-C), 62.9 (4’-C), 57.9 (11-C), 54.6 (10-C), 54.5 (2-C), 49.2 (13-C), 48.4 (2’’-C), 47.6 (8’’-C, 9’’-C), 44.5 (6’’-C), 38.1 (7-C), 36.4 (4’’-C, 10’’-C), 34.6 (5-C), 33.7 (1’’-C), 31.3 (5’’-C, 7’’-C), 27.7 (3’-C), 27.4 (1’-C), 24.8 (4-C), 24.4 (8-C), 21.9 (3-C), 18.3 (9-C), 18.1 (2’-C); HRMS *m/z*:Calcd for C_33_H_45_O_4_N_2_BrF_3_S [M–HCl+H]^+^, 701.2230; found, 701.2242; HPLC: *t_R_*=19.45min, purity 98.47%.

*12N-p-Trifluoromethlybenzenesulfonylmatrinicbutyl-3’-noradamantane-1’-carboxylate hydrochloride* (**9g**). Compound **5a** (0.50 g, 1.1 mmol) was treated with 3-noradamantanecarboxylic acid (**8d**, 0.17 g, 1.0 mmol) according to the general procedure to yield **9g** as a white solid, yield: 86%; m.p.: 186–187 °C; ^1^H NMR (600 MHz) δ 10.68 (s, 1H, 1-NH^+^), 8.07 (d, *J* = 8.1 Hz, 2H, 16-CH, 20-CH), 7.98 (d, *J* = 8.1 Hz, 2H, 17-CH, 19-CH), 4.22–4.15 (m, 1H, 11-CH), 3.88–3.81 (m, 2H, 13-CH_2_), 3.81–3.71 (m, 2H, 4’-CH_2_), 3.61–3.55 (m, 1H, 6-CH), 3.19 (d, *J* = 11.5 Hz, 2H, 2-CH_2_), 2.94–2.83 (m, 2H, 10-CH_2_), 2.48 (t, *J* = 6.8 Hz, 1H, 7’’-CH), 2.30–2.24 (m, 2H, 5-CH, 7-CH), 2.23–2.19 (m, 2H, 1’’-CH, 5’’-CH), 1.91–1.84 (m, 4H, 8-CH_2_, 2’’a-CH, 4’’a-CH), 1.70–1.48 (m, 16H, 4-CH_2_, 9-CH_2_, 1’-CH_2_, 2’-CH_2_, 2’’b-CH, 4’’b-CH, 6’’-CH_2_, 8’’-CH_2_, 9’’-CH_2_), 1.46–1.39 (m, 1H, 3’a-CH), 1.30–1.20 (m, 1H, 3’b-CH), 1.12–1.06 (m, 1H, 3a-CH), 0.91–0.82 (m, 1H, 3b-CH); ^13^C NMR (151 MHz) δ 176.3 (6’-C), 145.2 (15-C), 132.4 (18-C), 127.7 (17-C, 19-C), 126.6 (16-C, 20-C), 122.3 (21-C), 63.2 (6-C), 62.9 (4’-C), 58.0 (11-C), 54.6 (10-C), 54.5 (2-C), 53.2 (13-C), 48.6 (3’’-C), 46.2 (2’’-C, 4’’-C), 43.3 (9’’-C), 43.0 (6’’-C, 8’’-C), 38.2 (7-C), 36.8 (1’’-C, 5’’-C), 34.8 (5-C), 34.0 (7’’-C), 27.8 (3’-C), 27.3 (1’-C), 24.8 (4-C), 24.4 (8-C), 22.1 (3-C), 18.3 (9-C), 18.1 (2’-C); HRMS *m/z*:Calcd for C_32_H_44_O_4_N_2_F_3_S [M–HCl+H]^+^, 609.2968; found, 609.2970; HPLC: *t_R_*=18.69 min, purity 97.49%.

*12N-m-Trifluoromethlybenzenesulfonylmatrinicbutyl-3’-noradamantane-1’-carboxylate hydrochloride* (**9h**). Compound **5b** (0.50 g, 1.1 mmol) was treated with 3-noradamantanecarboxylic acid (**8d**, 0.17 g, 1.0 mmol) according to the general procedure to yield product **9h** as a white solid, yield: 84%; m.p.: 207 °C(dec.); ^1^H NMR (600 MHz) δ 10.74 (s, 1H, 1-NH^+^), 8.18 (d, *J* = 7.9 Hz, 1H, 18-CH), 8.07 (d, *J* = 7.8 Hz, 1H, 20-CH), 8.04 (s, 1H, 16-CH), 7.88 (t, *J* = 7.8 Hz, 1H, 19-CH), 4.22–4.15 (m, 1H, 11-CH), 3.88–3.81 (m, 2H, 13-CH_2_), 3.81–3.71 (m, 2H, 4’-CH_2_), 3.61–3.55 (m, 1H, 6-CH), 3.19 (d, *J* = 11.5 Hz, 2H, 2-CH_2_), 2.94–2.83 (m, 2H, 10-CH_2_), 2.48 (t, *J* = 6.8 Hz, 1H, 7’’-CH), 2.30–2.24 (m, 2H, 5-CH, 7-CH), 2.23–2.19 (m, 2H, 1’’-CH, 5’’-CH), 1.91–1.84 (m, 4H, 8-CH_2_, 2’’a-CH, 4’’a-CH), 1.70–1.48 (m, 16H, 4-CH_2_, 9-CH_2_, 1’-CH_2_, 2’-CH_2_, 2’’b-CH, 4’’b-CH, 6’’-CH_2_, 8’’-CH_2_, 9’’-CH_2_), 1.46–1.39 (m, 1H, 3’a-CH), 1.30–1.20 (m, 1H, 3’b-CH), 1.12–1.06 (m, 1H, 3a-CH), 0.91–0.82 (m, 1H, 3b-CH);^13^C NMR (151 MHz) δ 176.1 (6’-C), 143.0 (15-C), 131.2 (17-C), 130.8 (20-C), 129.9 (19-C), 129.5 (18-C), 122.9 (16-C), 122.3 (21-C), 63.2 (6-C), 62.9 (4’-C), 58.0 (11-C), 54.6 (10-C), 54.5 (2-C), 53.2 (13-C), 48.6 (3’’-C), 46.2 (2’’-C, 4’’-C), 43.3 (9’’-C), 43.0 (6’’-C, 8’’-C), 38.2 (7-C), 36.8 (1’’-C, 5’’-C), 34.8 (5-C), 34.0 (7’’-C), 27.8 (3’-C), 27.3 (1’-C), 24.8 (4-C), 24.4 (8-C), 22.1 (3-C), 18.3 (9-C), 18.1 (2’-C); HRMS *m/z*:Calcd for C_32_H_44_O_4_N_2_F_3_S [M–HCl+H]^+^, 609.2968; found, 609.2970; HPLC: *t_R_* =18.66 min, purity 96.80%.

*12N-p-Trifluoromethlybenzenesulfonyl matrinic butyl-2’-norbornane acetate hydrochloride*(**9i**). Compound **5a** (0.50 g, 1.1 mmol) was treated with 2-norbornaneacetic acid (**8e**, 0.15 g, 1.0 mmol) according to the general procedure to yield product **9i** as a white solid, yield: 73%; m.p.: 223 °C (dec.); ^1^H NMR (600 MHz) δ 10.58–10.49 (m, 1H, 1-NH^+^), 8.07 (d, *J* = 8.2 Hz, 2H, 16-CH, 20-CH), 7.98 (d, *J* = 8.2 Hz, 2H, 17-CH,19-CH), 4.09 (dt, *J* = 11.6, 5.8 Hz, 1H, 11-CH), 3.82–3.72 (m, 4H, 4’-CH_2_, 13-CH_2_), 3.59–3.55 (m, 1H, 6-CH), 3.20 (d, *J* = 11.8 Hz, 2H, 2-CH_2_), 2.93–2.85 (m, 2H, 10-CH_2_), 2.29–2.23 (m, 2H, 3’-CH_2_), 2.21–2.13 (m, 2H, 5-CH, 7-CH), 2.06–2.01 (m, 1H, 2’’-CH), 1.90–1.83 (m, 3H, 8-CH_2_, 5’’-CH), 1.76–1.71 (m, 1H, 1’’-CH), 1.71–1.54 (m, 8H, 4-CH_2_, 9-CH_2_,1’-CH_2_. 2’-CH_2_), 1.46–1.39 (m, 4H, 7’-CH_2_, 6’’-CH_2_), 1.28–1.22 (m, 2H, 3’’-CH_2_), 1.16–1.08 (m, 3H, 4’’-CH_2_, 7’’a-CH), 1.03 (d, *J* = 9.9 Hz, 1H, 7’’b-CH), 0.99–0.94 (m, 1H, 3a-CH), 0.92–0.86 (m, 1H, 3b-CH); ^13^C NMR (151 MHz) δ 172.0 (6’-C), 145.2 (15-C), 132.4 (18-C), 127.7 (16-C, 20-C), 126.6 (17-C, 19-C), 123.4 (21-C), 63.2 (4’-C), 62.8 (6-C), 57.7 (11-C), 54.6 (10-C), 54.5 (2-C), 48.4 (13-C), 40.5 (2’’-C, 5’’-C), 38.1 (7-C), 38.0 (1’’-C), 37.2 (7’-C), 36.1 (7’’-C), 34.7 (5-C), 34.6 (3’-C), 29.3 (6’’-C), 28.1 (3’’-C), 27.8 (4’’-C), 27.4 (1’-C), 24.8 (4-C), 24.4 (8-C), 21.9 (3-C), 18.3 (9-C), 18.1 (2’-C); HRMS *m/z*: Calcd for C_31_H_44_O_4_N_2_F_3_S [M–HCl+H]^+^, 597.2968; found, 531.2971; HPLC: *t_R_* = 18.31 min, purity 95.09%.

*12N-m-Trifluoromethlybenzenesulfonyl matrinic butyl-2’-norbornane acetate hydrochloride* (**9j**). Compound **5b** (0.50 g, 1.1 mmol) was treated with 2-norbornaneacetic acid (**8e**, 0.15 g, 1.0 mmol) according to the general procedure to yield product **9j** as a white solid, yield: 49%; m.p.: 85–86 °C; ^1^H NMR (600 MHz) δ 10.90 (q, *J* = 9.6 Hz, 1H, 1-NH^+^), 8.18 (d, *J* = 7.9 Hz, 1H, 16-CH), 8.08–8.02 (m, 2H, 18-CH, 20-CH), 7.87 (t, *J* = 7.9 Hz, 1H, 19-CH), 4.24 (t, *J* = 9.2 Hz, 1H, 11-CH), 3.91–3.87 (m, 1H,13a-CH), 3.84–3.78 (m, 1H,13b-CH), 3.71 (t, *J* = 6.7 Hz, 2H, 4’-CH_2_), 3.64–3.58 (m, 1H, 6-CH), 3.18 (d, *J* = 11.8 Hz, 2H, 2-CH_2_), 2.96–2.84 (m, 2H, 10-CH_2_), 2.31–2.27 (m, 2H, 5-CH, 7-CH), 2.20–2.11 (m, 2H, 3’-CH_2_), 2.05–1.98 (m, 1H, 2’’-CH), 1.95–1.85 (m, 3H, 8-CH_2_, 5’’-CH), 1.77–1.53 (m, 9H, 4-CH_2_, 9-CH_2_,1’-CH_2_. 2’-CH_2_, 1’’-CH), 1.47–1.37 (m, 4H, 7’-CH_2_, 6’’-CH_2_), 1.27–1.20 (m, 2H, 3’’-CH_2_), 1.15–0.93 (m, 5H, 4’’-CH_2_, 7’’-CH_2_,3a-CH), 0.86–0.78 (m, 1H, 3b-CH); ^13^C NMR (151 MHz) δ 172.0 (6’-C), 143.1 (15-C), 131.1 (17-C), 130.7 (20-C), 129.9 (19-C), 129.4 (18-C), 122.8 (21-C), 122.6 (16-C), 63.1 (4’-C), 63.0 (6-C), 58.2 (11-C), 54.6 (10-C), 54.5 (2-C), 48.8 (13-C), 40.5 (2’’-C, 5’’-C), 38.2 (7-C), 37.9 (1’’-C), 37.1 (7’-C), 36.1 (7’’-C), 35.0 (5-C), 34.7 (3’-C), 29.3 (6’’-C), 28.1 (3’’-C), 27.7 (4’’-C), 27.2 (1’-C), 24.8 (4-C), 24.3 (8-C), 22.3 (3-C), 18.3 (9-C), 18.0 (2’-C); HRMS *m/z*: Calcd for C_31_H_44_O_4_N_2_F_3_S [M–HCl+H]^+^, 597.2968; found, 597.2978; HPLC: *t_R_*= 17.89 min, purity 96.83%.

#### 3.1.3. General Procedure for the Synthesis of Compounds **9k–l**

Trimethyl acetic anhydride **8f** (1.2 mmol) was dissolved in CH_2_Cl_2_ (10 mL), then it was added into a solution of **5a−b** (1.1 mmol) and TEA (1.5 mmol) in CH_2_Cl_2_ (30 mL), and the reaction solution was stirred at r.t. until TLC analysis showed completion of the reaction. The solution was washed by saturated aqueous sodium carbonate (50 mL), saturated aqueous ammonium chloride (50 mL × 2), saturated aqueous sodium chloride (50 mL), dried over anhydrous Na_2_SO_4_, and concentrated under reduced pressure. The residue was purified by flash column chromatography on silica gel with CH_2_Cl_2_/CH_3_OH as the eluent, and treated with 2 N HCl/ether (3 mL) to yield the desired products. Their structures and position numbers are shown in Figure 5. ^1^H NMR and ^13^CNMR spectrum of the compound **9k**-**l** see Supplementary Materials.

*12N-p-Trifluoromethlybenzenesulfonyl matrinic butyl pivalate hydrochloride* (**9k**). Compound **5a** (0.50 g, 1.1 mmol) was treated according to the general procedure to give the product **9k** as a white solid, yield: 78%; m.p.: 219–221 °C; ^1^H NMR (600 MHz) δ 10.62 (s, 1H,1-NH^+^), 8.07 (d, *J* = 8.2 Hz, 2H,16-CH, 20-CH), 7.98 (d, *J* = 8.2 Hz, 2H, 17-CH, 20-CH), 4.15–4.06 (m, 1H, 11-CH), 3.83–3.73 (m, 4H, 13-CH_2_, 4’-CH_2_), 3.59–3.54 (m, 1H, 6-CH), 3.19 (d, *J* = 12.0 Hz, 2H, 2-CH_2_), 2.93–2.84 (m, 2H, 10-CH_2_), 2.30–2.21 (m, 2H, 5-CH, 7-CH), 1.88–1.84 (m, 2H, 8-CH_2_), 1.68–1.54 (m, 9H, 4-CH_2_, 9-CH_2_,1’-CH_2_, 2’-CH_2_,3’a-CH), 1.46–1.42 (m, 1H, 3’b-CH), 1.26–1.23 (m, 1H, 3a-CH), 1.08 (s, 9H, 8’-CH_3_, 9’-CH_3_, 10’-CH_3_), 0.89–0.80 (m, 1H, 3b-CH); ^13^C NMR (151 MHz) δ 177.2 (6’-C), 145.2 (15-C), 132.5 (18-C), 127.7 (16-C, 20-C), 126.5 (17-C, 19-C), 123.4 (21-C), 63.4 (6-C), 62.9 (4’-C), 57.7 (11-C), 54.6 (10-C), 54.5 (2-C), 48.5 (13-C), 38.1 (7-C), 38.0 (7’-C), 34.6 (5-C), 27.8 (3’-C), 27.4 (1’-C), 26.8 (8’-C, 9’-C, 10’-C), 24.8 (4-C), 24.3 (8-C), 21.7 (3-C), 18.3 (9-C), 18.1 (2’-C); HRMS *m/z*: Calcd for C_27_H_40_O_4_N_2_F_3_S [M–HCl+H]^+^, 545.2655; found, 545.2645; HPLC: *t_R_* = 16.32 min, purity 97.21%.

*12N-m-Trifluoromethlybenzenesulfonyl matrinicbutylpivalate hydrochloride* (**9l**). Compound **5b** (0.50 g, 1.1 mmol) was treated according to the general procedure to yield product **9l** as a white solid, yield: 74%; m.p.: 162–164°C; ^1^H NMR (600 MHz) δ 10.69 (s, 1H, 1-NH^+^), 8.18 (d, *J* = 8.2 Hz, 1H, 16-CH), 8.07 (d, *J* = 7.9 Hz, 1H, 18-CH), 8.04 (s, 1H, 20-CH), 7.88 (t, *J* = 7.9 Hz, 1H, 19-CH), 4.21–4.15 (m, 1H, 11-CH), 3.87–3.78 (m, 2H, 13-CH_2_), 3.76–3.69 (m, 2H, 4’-CH_2_), 3.61–3.55 (m, 1H, 6-CH), 3.19 (d, *J* = 11.8 Hz, 2H, 2-CH_2_), 2.93–2.84 (m, 2H, 10-CH_2_), 2.31–2.24 (m, 2H, 5-CH, 7-CH), 1.90–1.86 (m, 2H, 8-CH_2_), 1.73–1.51 (m, 9H, 4-CH_2_, 9-CH_2_,1’-CH_2_, 2’-CH_2_,3’a-CH), 1.46–1.38 (m, 1H, 3’b-CH), 1.28–1.19 (m, 1H, 3a-CH), 1.07 (s, 9H, 8’-CH_3_, 9’-CH_3_, 10’-CH_3_), 0.89–0.80 (m, 1H, 3b-CH); ^13^C NMR (151 MHz) δ 177.2 (6’-C), 143.0 (15-C), 131.2 (17-C), 130.8 (20-C), 129.9 (19-C), 129.5 (18-C), 123.6 (21-C), 122.9 (16-C), 63.3 (6-C), 62.9 (4’-C), 58.0 (11-C), 54.6 (10-C), 54.5 (2-C), 48.6 (13-C), 38.2 (7-C), 38.1 (7’-C), 34.8 (5-C), 27.8 (3’-C), 27.3 (1’-C), 26.8 (8’-C, 9’-C, 10’-C), 24.8 (4-C), 24.4 (8-C), 22.1 (3-C), 18.3 (9-C), 18.1 (2’-C); HRMS *m/z*:Calcd for C_27_H_40_O_4_N_2_F_3_S [M–HCl+H]^+^, 545.2655; found, 545.2656; HPLC: *t_R_*= 16.14 min, purity 97.70%.

### 3.2. Biology

#### 3.2.1. Anti–H3N2 and Anti–H1N1 Effects In Vitro

Influenzavirus A/Fort Monmouth/1/1947 (H1N1) and MDCK cells were purchased from ATCC (the American Type Culture Collection). Influenza virus A/hanfang/359/95 (H3N2) was kindly donated by Professor Yuelong Shu from the Institute for Viral Disease Control and Prevention, China Centers for Disease Control and Prevention, Beijing, China. Each compound was dissolved in DMSO at an initial concentration of 20 mg/mL, and then diluted three-fold successively to obtain eight different concentrations as stock solutions for the following experiments. MDCK cells were seeded in 96-well trays, and cultured at 37 °C in a humidified CO_2_ incubator (95% air, 5% CO_2_) for 24 hr. Then, the cells were infected with influenza A virus. All infected tissue culture plates (96 wells) were incubated at 37 °C for 2 hr, and then the medium was removed. Subsequently, different concentrations of drug maintenance solutions were added to the wells (one per well), and the plates were incubated again for 40 hr at 37 °C. Then, the inhibition of the virus-induced cytopathic effect (CPE) for each sample was recorded relative to the cell control and the virus control. The IC_50_ value of each compound was calculated accordingly [22]. The scoring criteria of CPE was as follows: (0) no CPE; (1) 0~25% of cells appeared CPE; (2) 25%~50% of cells appeared CPE; (3) 50%~75% of the cells appeared CPE; (4) 75% to 100% of cells appeared CPE. According to the references, the 50% CPE inhibition concentrations (IC_50_) of compounds were determined using the Reed and Muench method [23].

#### 3.2.2. Cytotoxicity Assay

MDCK cells were seeded in 96-well trays; each well contained 25,000 cells and was cultured at 37 °C in a humidified CO_2_ incubator (95% air, 5% CO_2_) for 24 hr. A three-fold dilution compound was added to cell monolayer, and the cells continued to be cultivated for 48 hr, and then the CPE was recorded [21]. The TC_50_ value of each compound was calculated by the methods of Reed & Muench.

#### 3.2.3. Stability Assay of Key Compounds in Whole Blood

The fresh bloods were collected on the day of experiment from SD rats, and pre-warmed in a water bath at 37 °C. A concentration of 10 mM tested compound or enalapril stock solutions were prepared in DMSO, and then diluted with 45% MeOH/H_2_O to achieve 100 mM dosing solutions. Each dosing solution (2 mL) was incubated with 98 mL of blank blood at 37 °C in water bath for 0, 1, 5, 10, 20, 40, 60, and 120 min, respectively. At the end of incubation, for each sample, 100 mL water and 800 mL of stop solution (200 ng·mL^−1^ tolbutamide plus 20 ng·mL^−1^ buspirone in acetonitrile) were immediately added to precipitate the protein, and centrifuged at 4000 rpm for 20 min. An aliquot of supernatant (100 mL) was then extracted, mixed with 200 mL H_2_O, and then shaken at 800 rpm for about 10 min before being submitted for a liquid chromatography-tandem mass chromatography (LC-MS/MS) analysis. The experiment was repeated twice.

#### 3.2.4. Acute Toxicity Assay

Female Kunming mice with weights of 20.0 (±1.0 g) were fed with regular rodent chow, and housed in an air-conditioned room. The mice were randomly divided into different groups with 10 mice each. Each compound was given orally in a single-dosing experiment at 125, 250, or 500 mg·kg^−1^, respectively. The mice were closely monitored for seven days. Body weight, as well as survival, was monitored.

#### 3.2.5. Anti–H3N2 Activity Assay at the Protein Level

MDCK cells were plated into 6-well culture plates for incubation of 16 hr. The medium was removed, and cells were infected with influenza virus A/hanfang/359/95(H3N2) at a MOI of 0.003 for 2 hr. Then, various concentrations of the tested compounds were supplemented immediately for incubation for another 24 hr. The cells were harvested for Western blot assay. Proteins were detected using antibodies directed against β-actin (1:5000) (Cell Signaling Technology, Beverly, MA, USA), IAV M2 and NS1 (1:400) (Santa Cruz, Dallas, TX, USA), respectively.

#### 3.2.6. Time-of-Addition Assay

MDCK cells were infected with influenza virus A/hanfang/359/95 (H3N2) (MOI=0.03) and simultaneously treated with compound or solvent control. After being treated with indicated time (−2 h, 0 h, 2 h, 4 h, and 6 h, respectively), the cells were washed with PBS, and fresh cultural media were added, to continuously incubate the cells. Total intracellular proteins were extracted with lysis buffer at 8 hr p.i., and detected by Western blot.

## 4. Conclusions

A series of novel tricyclic matrinic derivatives with an 11-rigid group were designed, synthesized, and evaluated for their activities against influenza virus A H3N2, based on the privileged structure strategy. SAR analysis indicated that the introduction of an 11-adamantyl or norbornanecetyl might be helpful for the activity. Among them, compounds **9f** and **9j** exhibited promising anti-H3N2 activities, with IC_50_ values of 7.2 μM and 10.2 μM, respectively, which were significantly higher than that of lead **1**, but weaker than that of parent amantadine. Their activities were further confirmed at the protein level. Moreover, compound **9f** also displayed a high PK stability profile in whole blood, and a safety profile in vivo. In the primary mechanism, compound **9f** could inhibit the virus replication cycle at an early stage by targeting the M2 protein, which was consistent with that of amantadine. This study provided powerful information for further strategic optimization of its kind, as a new class of anti-influenza agents.

## Figures and Tables

**Table 1:**
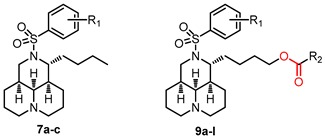
Anti-H3N2 activities of all title compounds and anti-H1N1 activities of key compounds.

Compound	R_1_	R_2_	TC_50_ (µM) ^a^	H3N2, A/hanfang/359/95		H1N1, A/Fort Monmouth/1/1947	
				IC_50_ (µM) ^b^	SI ^c^	IC_50_ (µM)	SI
**1**			>540.5	16.2	> 33.4	NT ^d^	NT
**7a**	*p*-CF_3_	CH_3_	79.8 ± 4.7	21.3 ± 1.9	3.8	NT	NT
**7b**	*m*-CF_3_	CH_3_	86.6 ± 2.3	28.9 ± 2.4	3.0	NT	NT
**7c**	*o*-CF_3_	CH_3_	86.6 ± 3.8	50.0 ± 1.1	1.7	NT	NT
**9a**	*p*-CF_3_	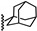	19.5 ± 2.8	16.5 ± 2.9	1.2	NT	NT
**9b**	*p*-CF_3_	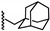	61.9 ± 1.9	28.4 ± 2.3	2.2	NT	NT
**9c**	*p*-CF_3_	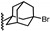	73.6 ± 4.3	8.2 ± 2.07	9.0	27.5 ± 0.1	2.7
**9d**	*m*-CF_3_	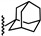	54.9 ± 3.5	5.9 ± 0.9	9.2	22.4 ± 1.3	2.5
**9e**	*m*-CF_3_	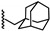	63.4 ± 2.3	11.1 ± 1.5	5.7	NT	NT
**9f**	*m*-CF_3_	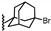	75.7 ± 14.2	7.2 ± 1.1	10.6	23.4 ± 1.5	3.2
**9g**	*p*-CF_3_	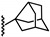	38.5 ± 6.6	– ^e^	–	NT	NT
**9h**	*m*-CF_3_	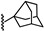	59.7 ± 0.0	–	–	NT	NT
**9i**	*p*-CF_3_	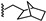	129.0 ± 20.9	25.7 ± 4.8	5.0	NT	NT
**9j**	*m*-CF_3_	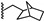	110.3 ± 18.5	10.2 ± 1.8	10.8	22.6 ± 1.3	4.9
**9k**	*p*-CF_3_	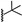	94.3 ± 11.3	–	–	NT	NT
**9l**	*m*-CF_3_	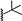	52.9 ± 8.0	18.2 ± 3.2	2.9	NT	NT
**AH**			>200	3.25 ± 0.5	>61.5	2.73	>73.3

^a^ 50% cytotoxic concentration (µM); ^b^ 50% virus–inhibitory concentration (µM) after being incubated for 40 hr at 37 °C; ^c^ selectivity index (SI) values equaled to TC_50_/IC_50_; ^d^ not tested; ^e^ no anti–H3N2 activity at themaximum non-toxic concentration.

## Supplementary Materials

The following are available online at https://www.mdpi.com/1420-3049/24/5/921/s1, Figure S1–S24: ^1^H NMR and ^13^CNMR spectrum of all new compounds **9a**–**9l**.
