# Supplementary material for: Evolution and Biological Evaluation of Matrinic Derivatives with Amantadine Fragments As New Anti-Influenza Virus Agents
Source: Molecules. 2019 Mar 6;24(5):921. doi: 10.3390/molecules24050921 (PMC6429159; doi:10.3390/molecules24050921)
Supplement: Supplementary file 1 [file molecules-24-00921-s001.pdf]

Article

Evolution and Biological Evaluation of Matrinic Derivatives with Amantadine Fragments As New Anti-Influenza Virus Agents

Tianyu Niu [†], Xiaoqiang Zhao [†], Jing Jiang, Haiyan Yan, Yinghong Li, Sheng Tang *, Yuhuan Li * and Danqing Song

Beijing Key Laboratory of Anti-infective Agents, Institute of Medicinal Biotechnology, Chinese Academy of Medical Science & Peking Union Medical College, Beijing, 10005, China;niutianyubasn@163.com (T.N.); xiaoqiangzhao@126.com (X.Z.); 18299093210@163.com(J.J); yan0495@163.com(H.Y.); liyinghong@imb.pumc.edu.cn (Y.L.); songdanqingsdq@hotmail.com (D.S.)

* Correspondence: tang13874204108@163.com (S.T.); yuhuanlibj@126.com (Y.L.); Tel.: +86-106-303-3012 (S.T); Tel.: +86-106-303-3012 (Y.L.)

† These authors contribute equally to this work.

Figure 1S-24S: ^{1}H NMR and ^{13}CNMR spectrum of all new compounds 9a—9l

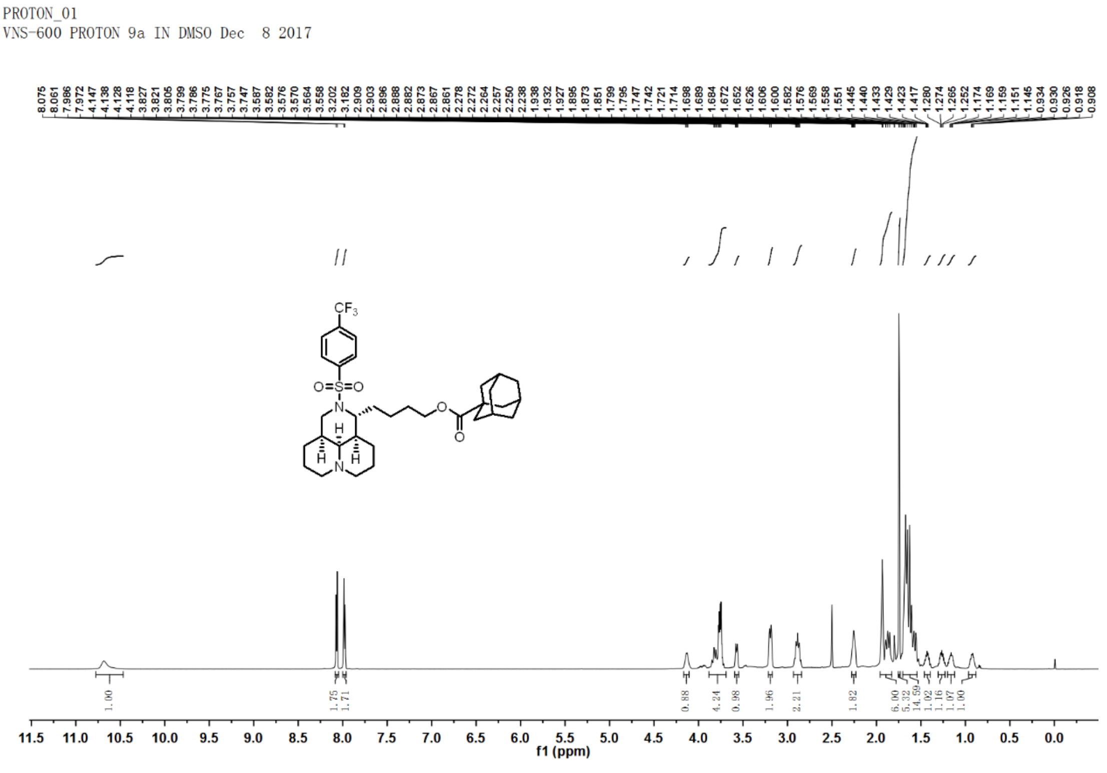

Figure 1S. ^{1}H NMR spectrum of compound of 9a

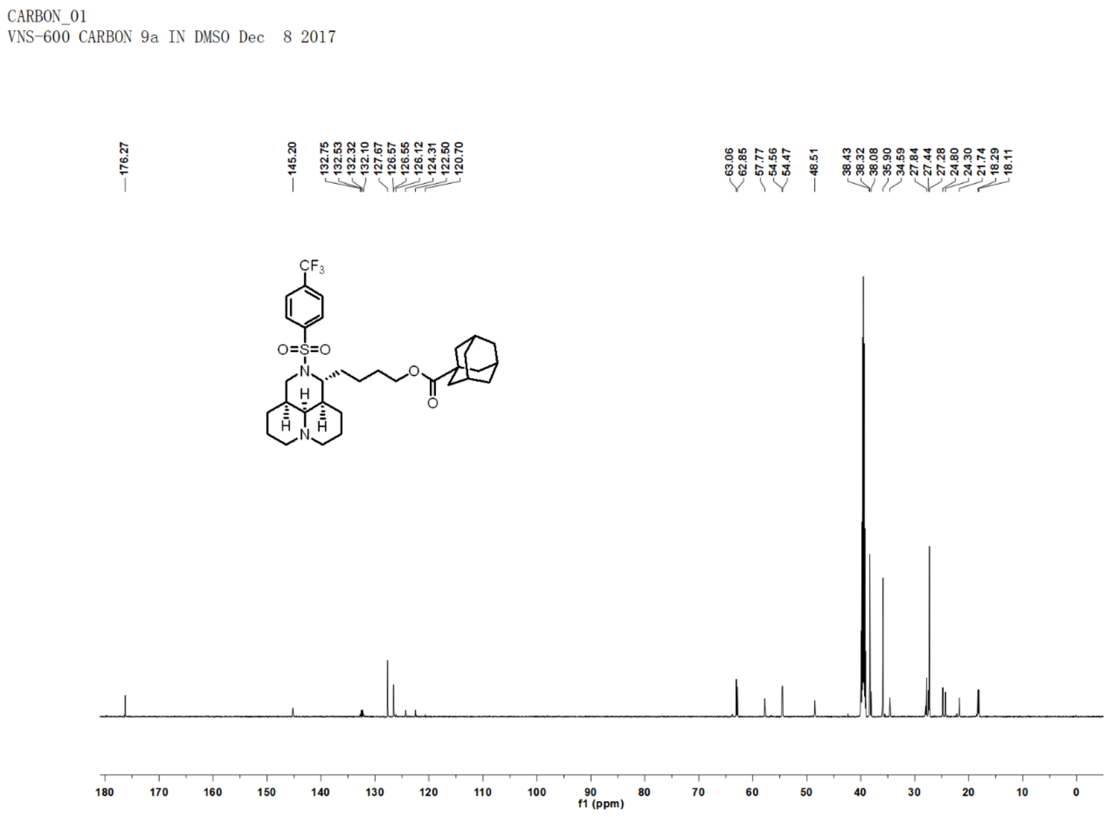

Figure 2S. ^{13}C NMR spectrum of compound of 9a

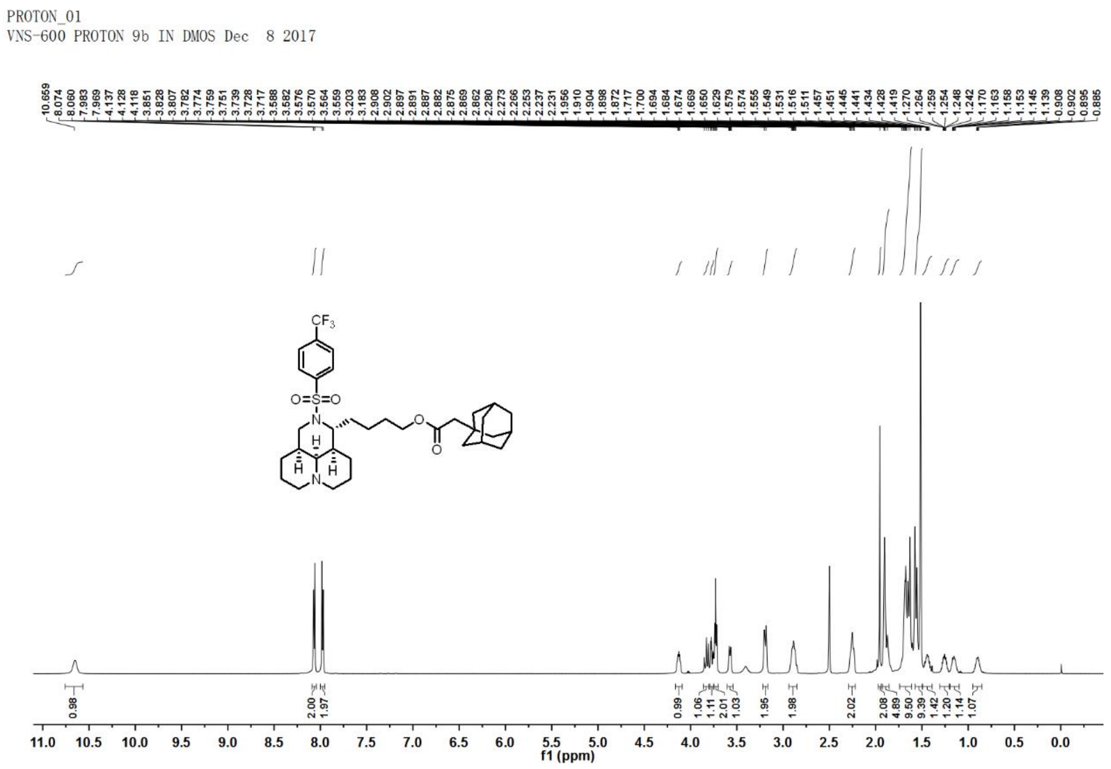

Figure 3S. ^{1}H NMR spectrum of compound of 9b

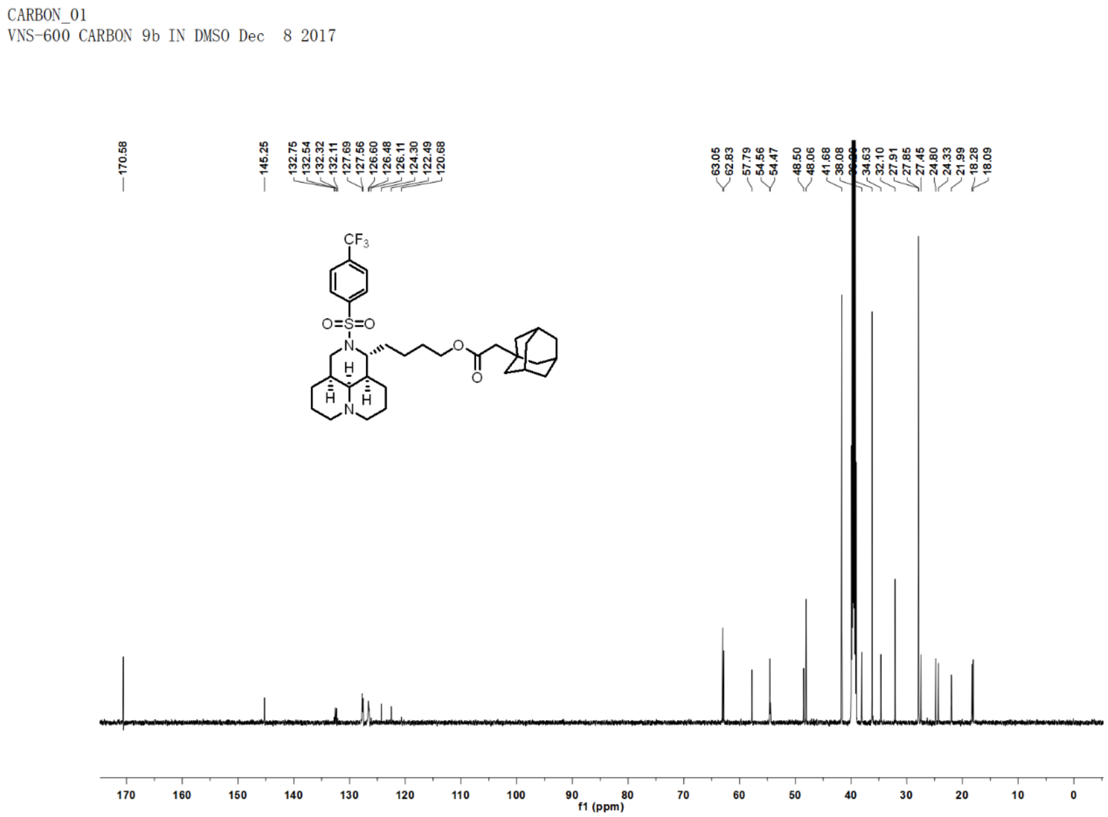

Figure 4S. ^{13}C NMR spectrum of compound of 9b

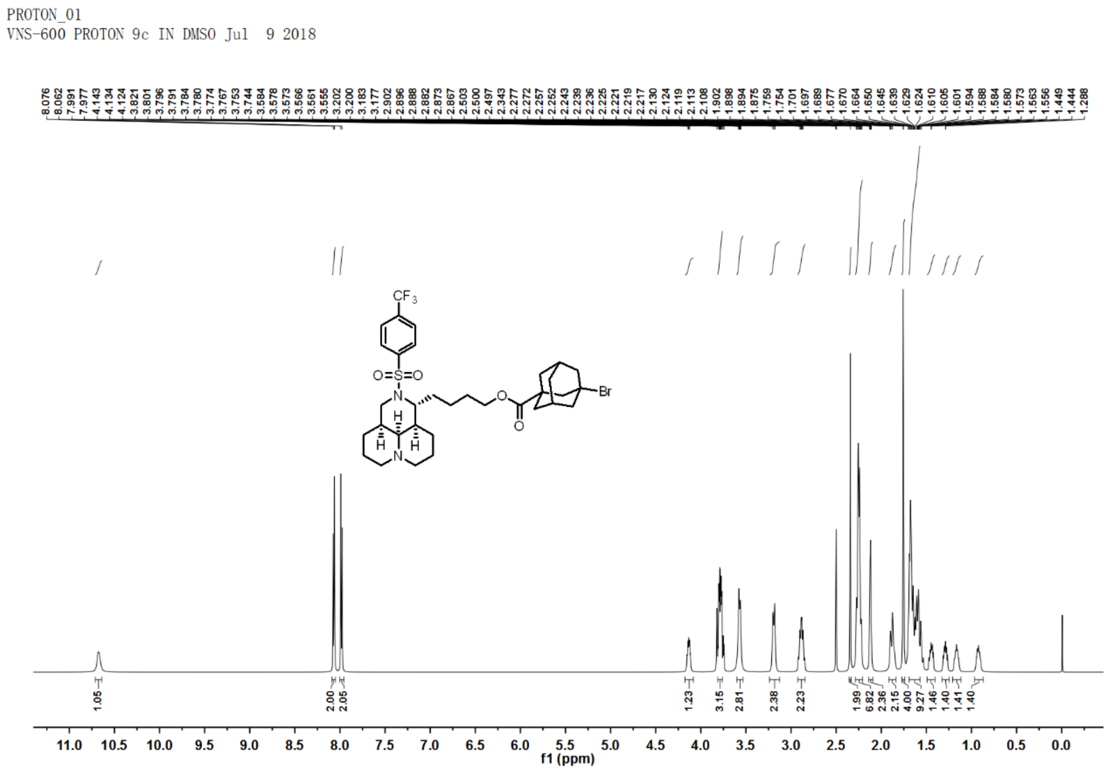

Figure 5S. ^{1}H NMR spectrum of compound of 9c

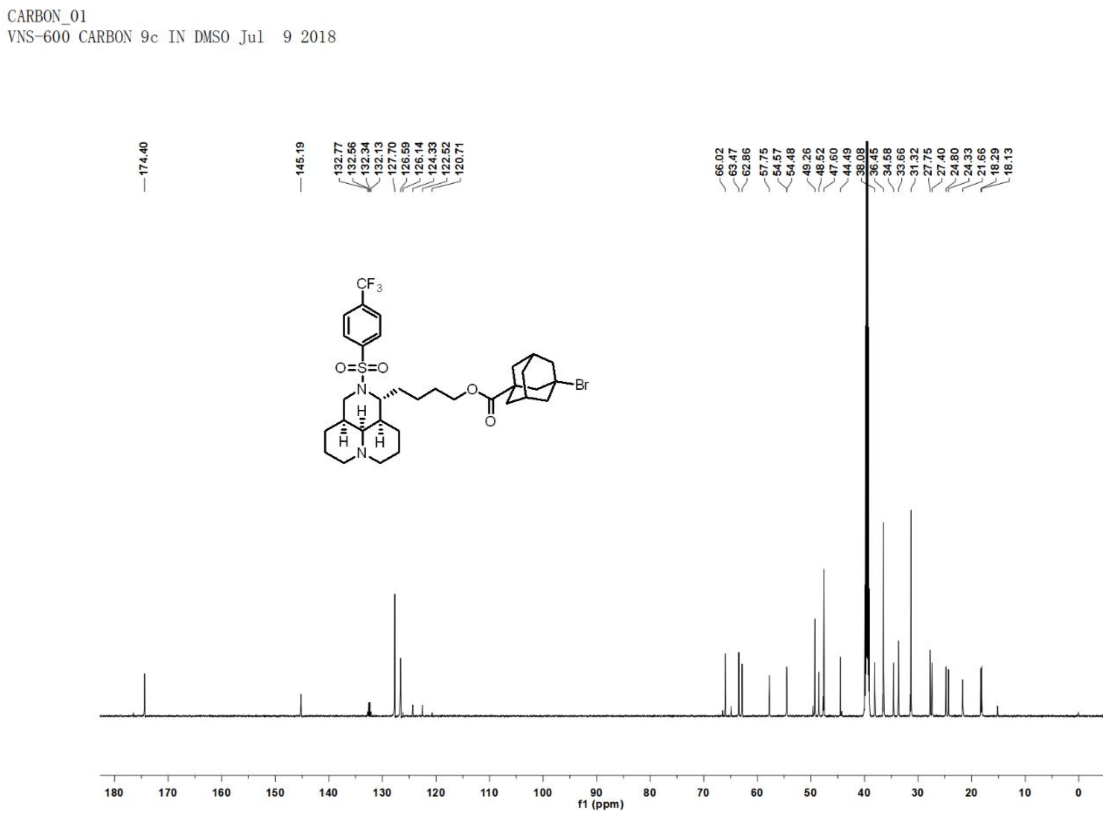

Figure 6S. ^{13}C NMR spectrum of compound of 9c

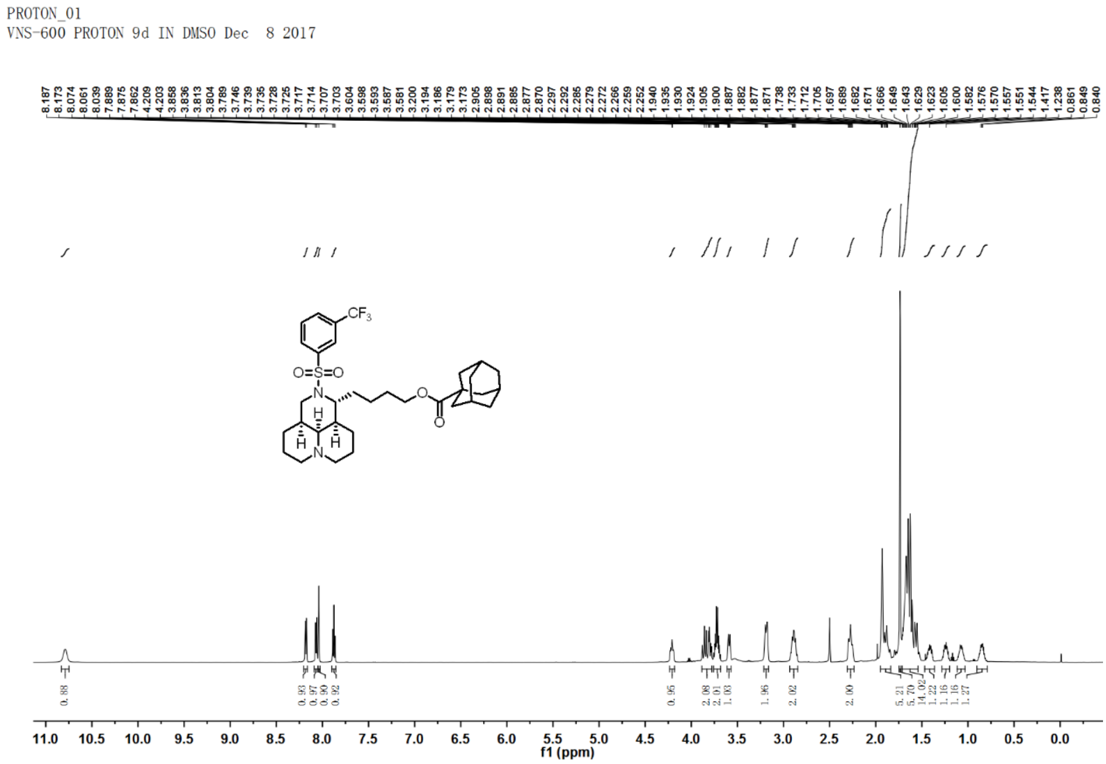

Figure 7S. ^{1}H NMR spectrum of compound of 9d

CARBON_01
VNS-600 CARBON 9d IN DMSO Dec 8 2017

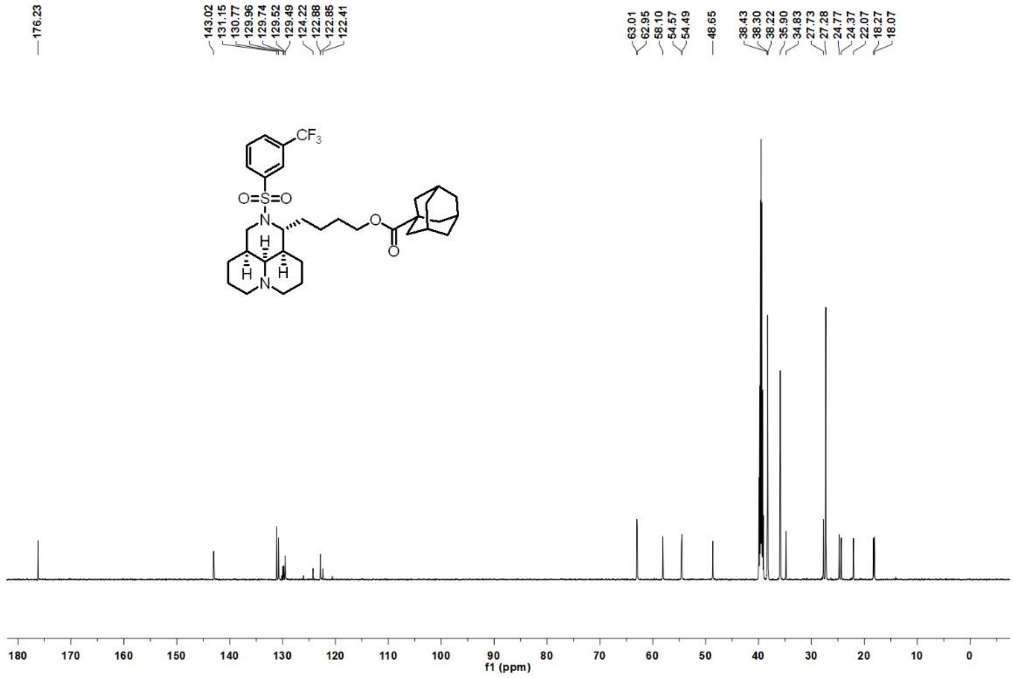

Figure 8S. ^{13}C NMR spectrum of compound of 9d

PROTON_01
VNS-600 PROTON 9e IN DMSO Dec 8 2017

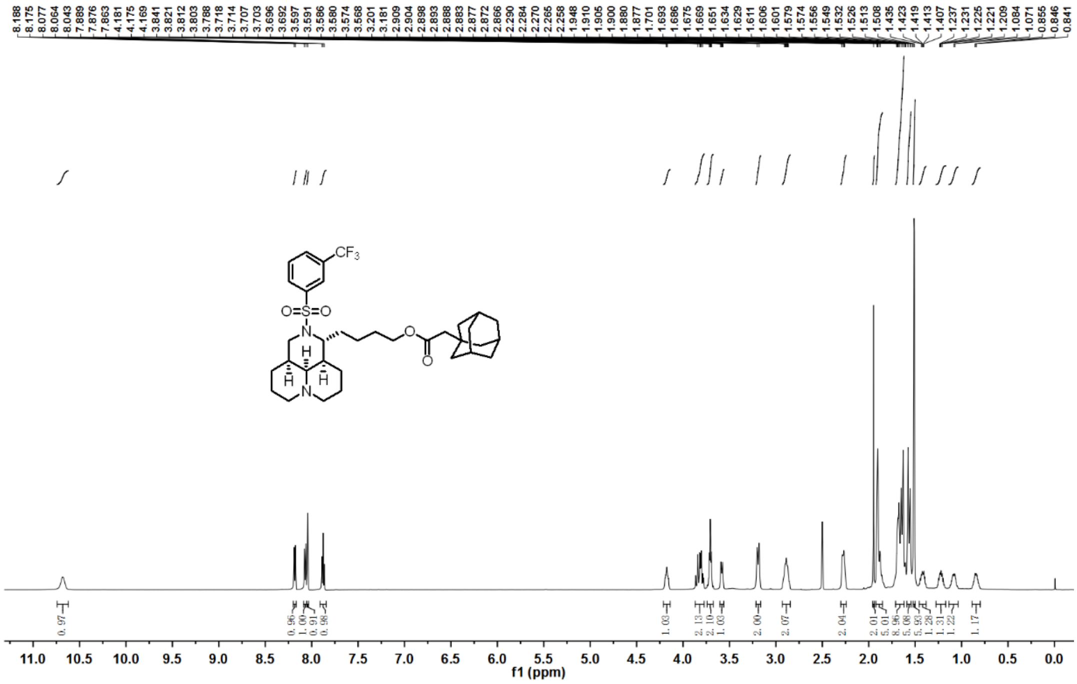

Figure 9S. ^{1}H NMR spectrum of compound of 9e

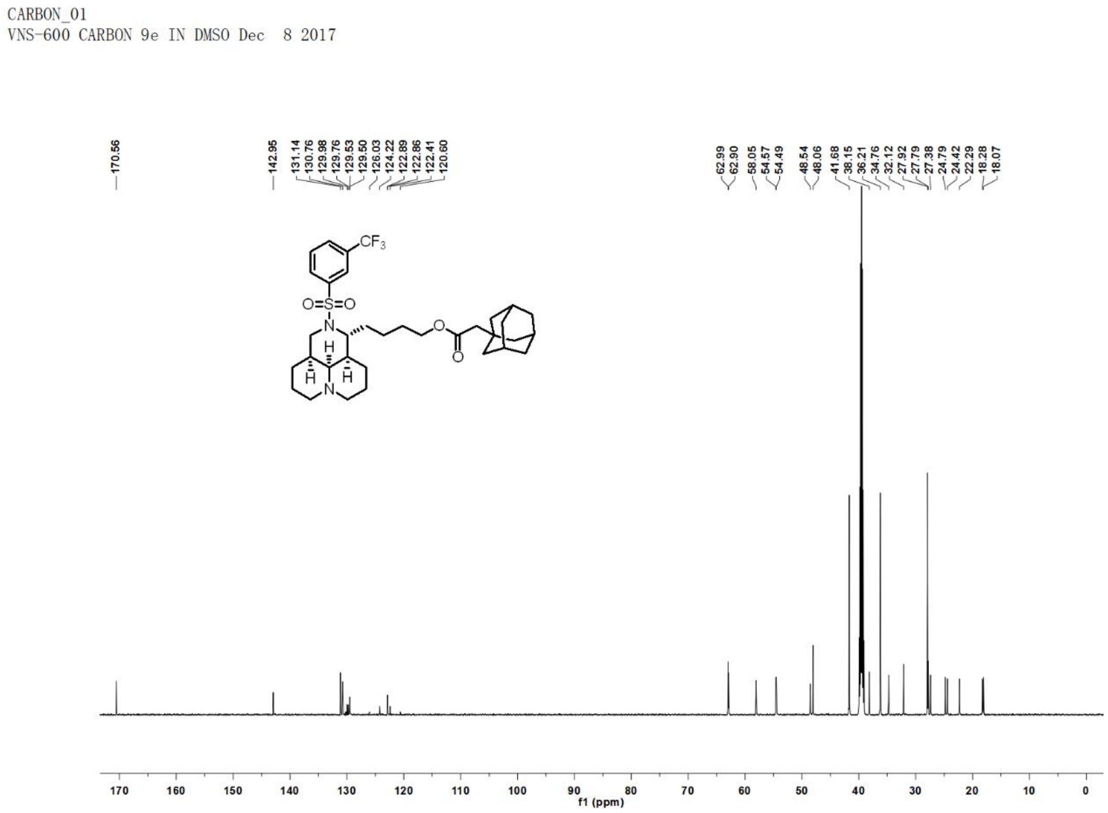

Figure 10S. ^{13}C **NMR spectrum of compound of 9e**

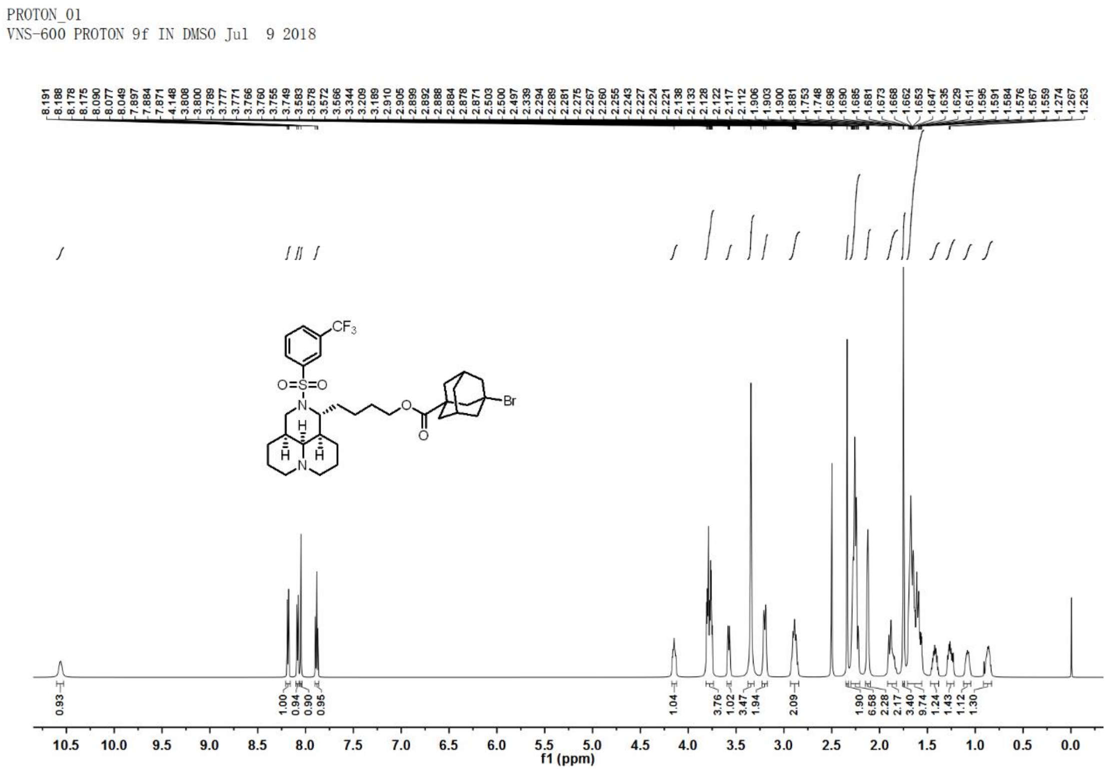

Figure 11S. ^{1}H **NMR spectrum of compound of 9f**

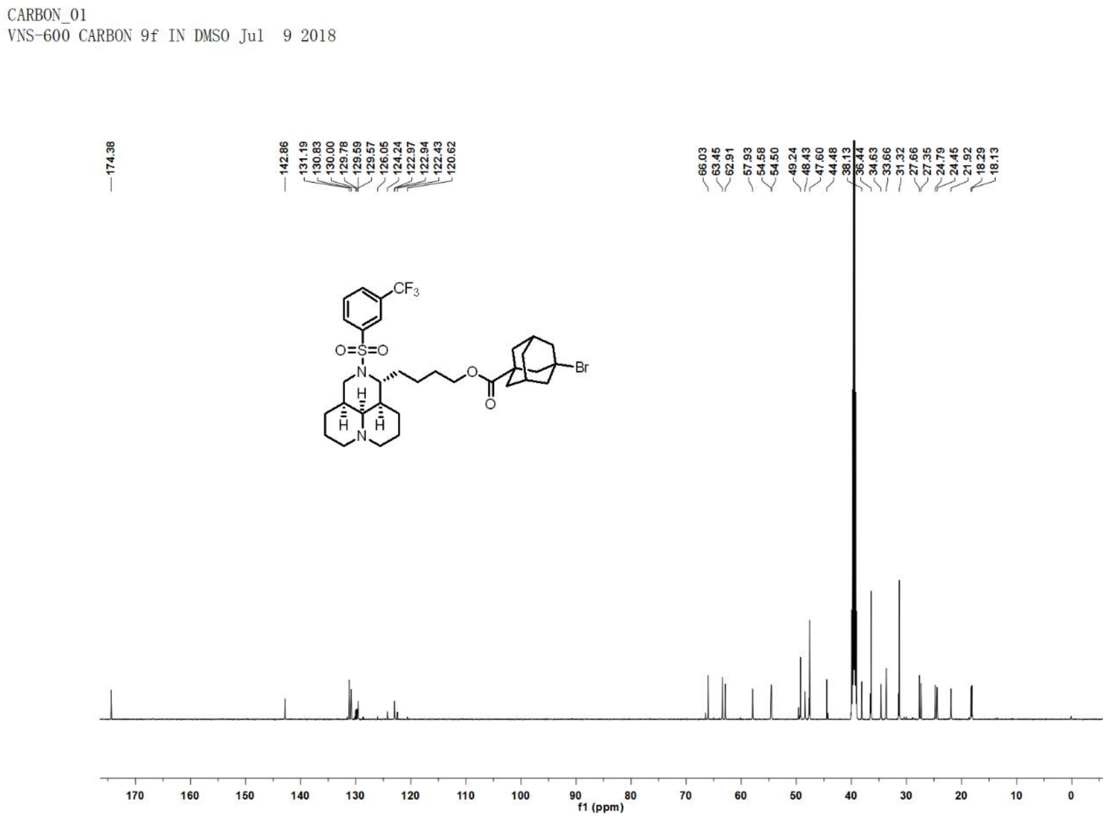

Figure 12S. ^{13}**C NMR spectrum of compound of 9f**

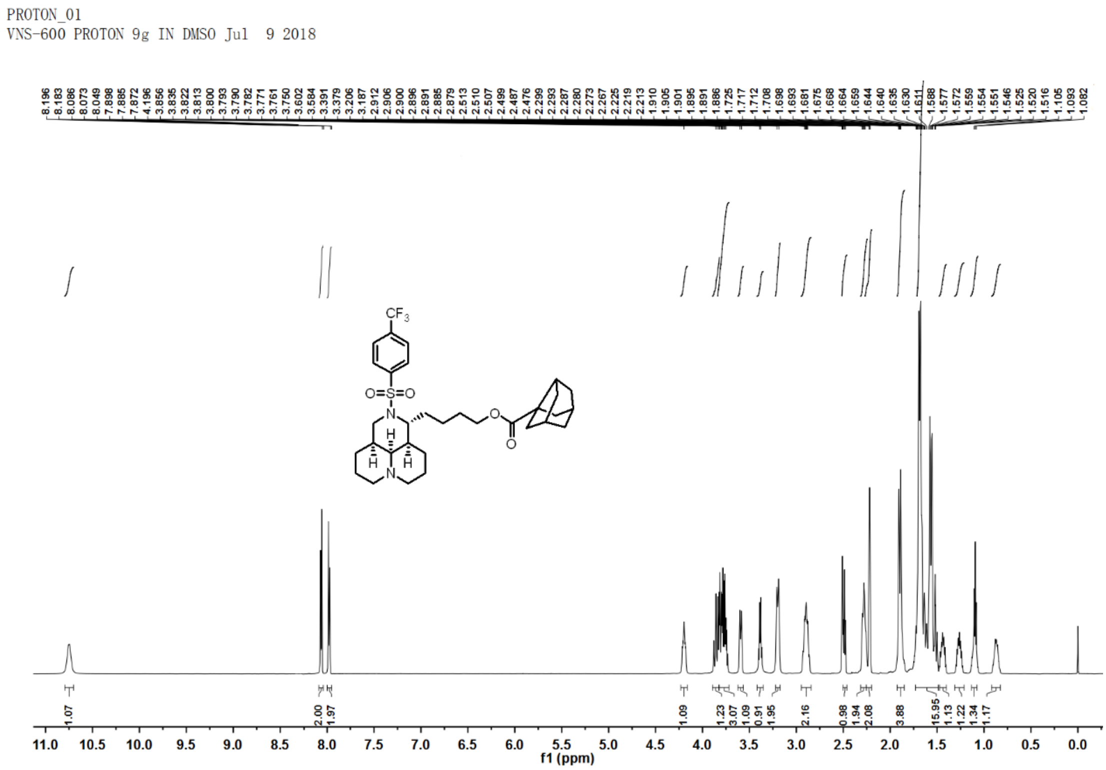

Figure 13S. ^{1}**H NMR spectrum of compound of 9g**

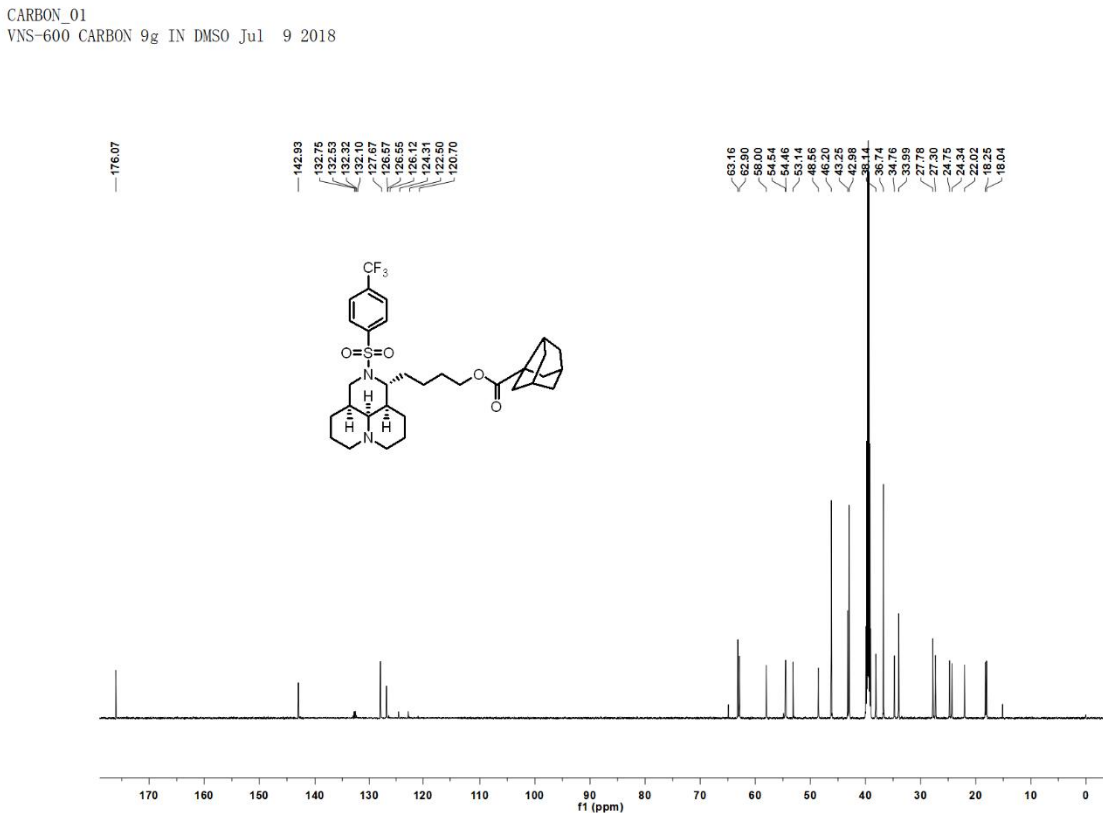

Figure 14S. ^{13}C **NMR spectrum of compound of 9g**

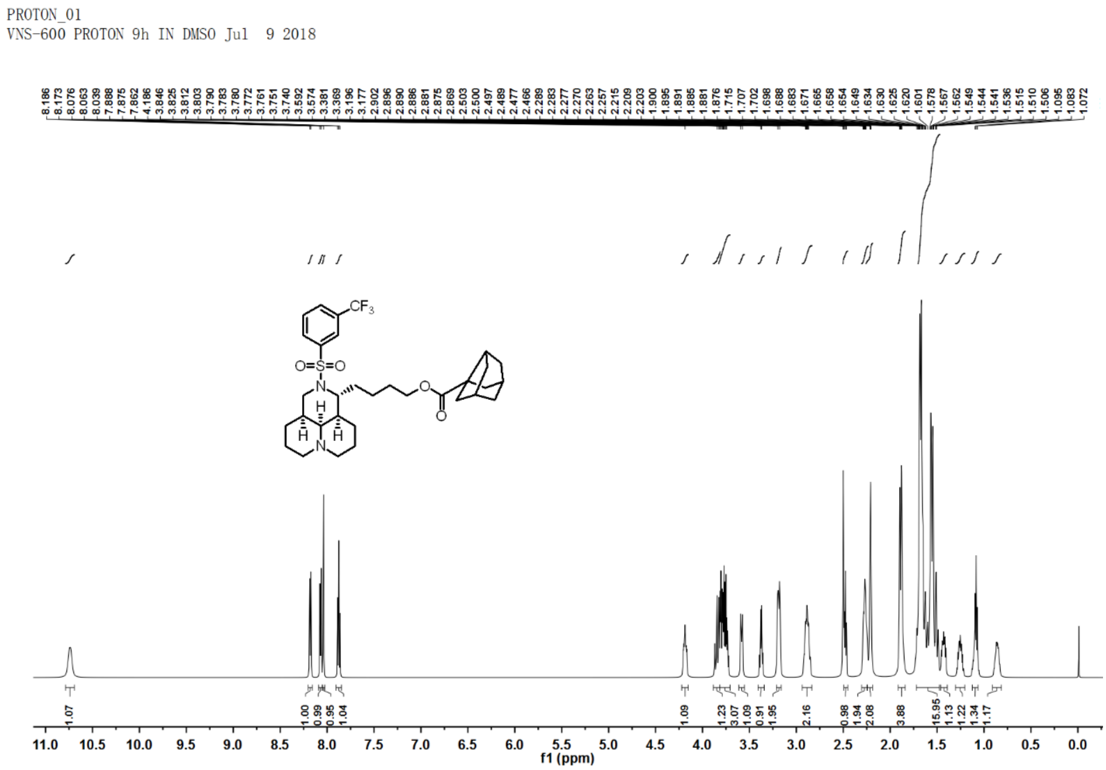

Figure 15S. ^{1}H **NMR spectrum of compound of 9h**

CARBON_01
VNS-600 CARBON 9h IN DMSO Jul 9 2018

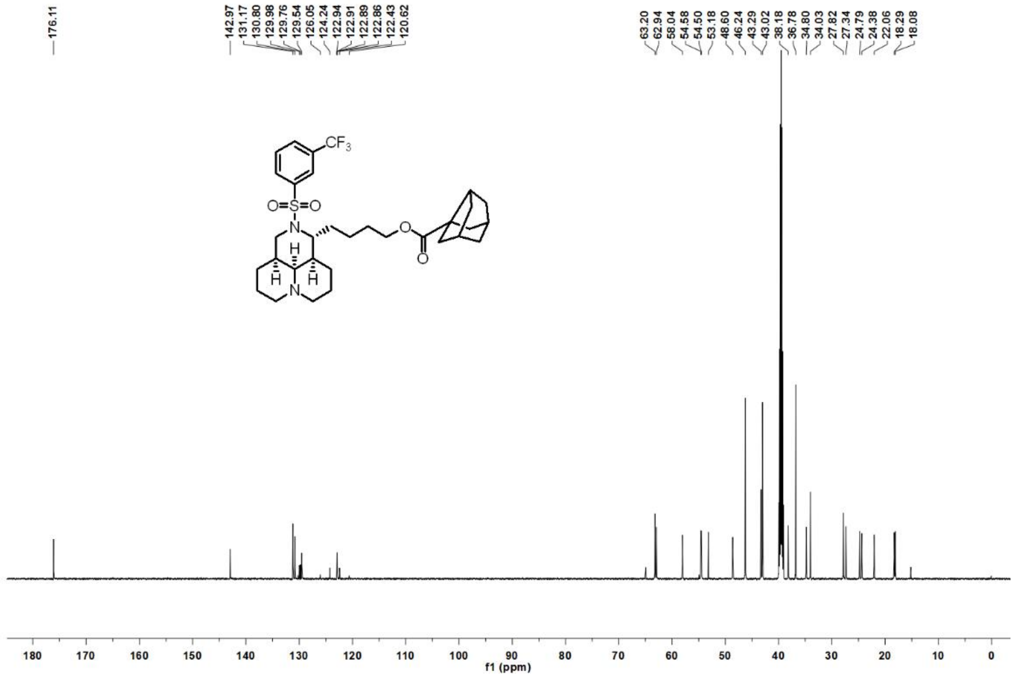

Figure 16S. ^{13}C NMR spectrum of compound of 9h

PROTON_01
VNS-600 PROTON 9i IN DMSO Dec 6 2017

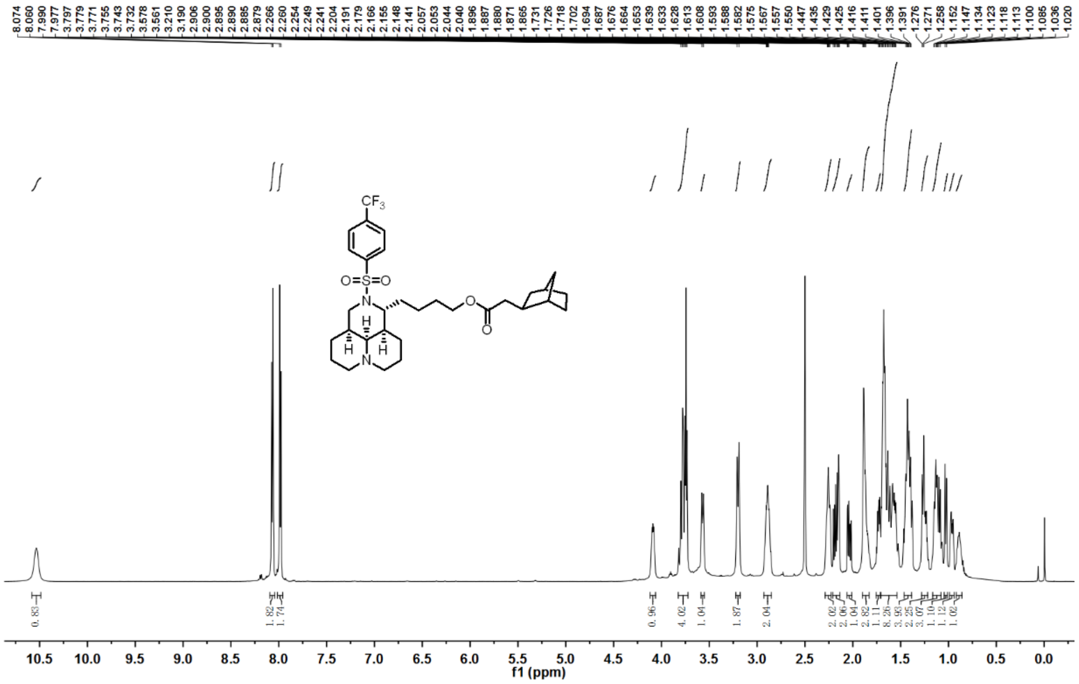

Figure 17S. ^{1}H NMR spectrum of compound of 9i

CARBON_01
VNS-600 CARBON 9i IN DMSO Dec 6 2017

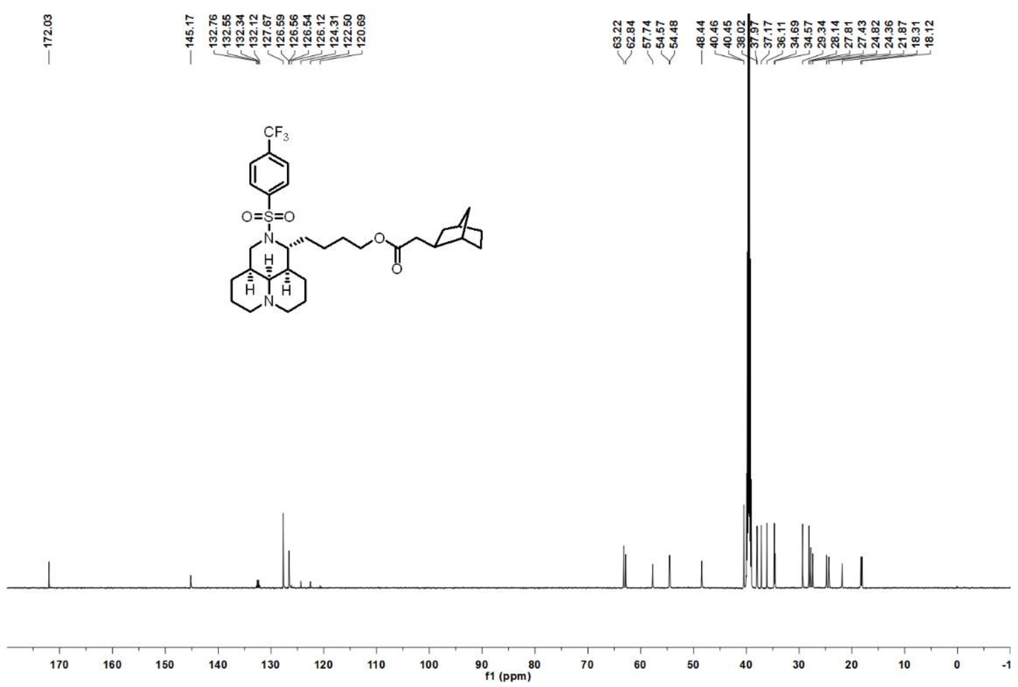

Figure 18S. ^{13}C NMR spectrum of compound of 9i

PROTON_01
VNS-600 PROTON 9j IN DMSO Dec 1 2017

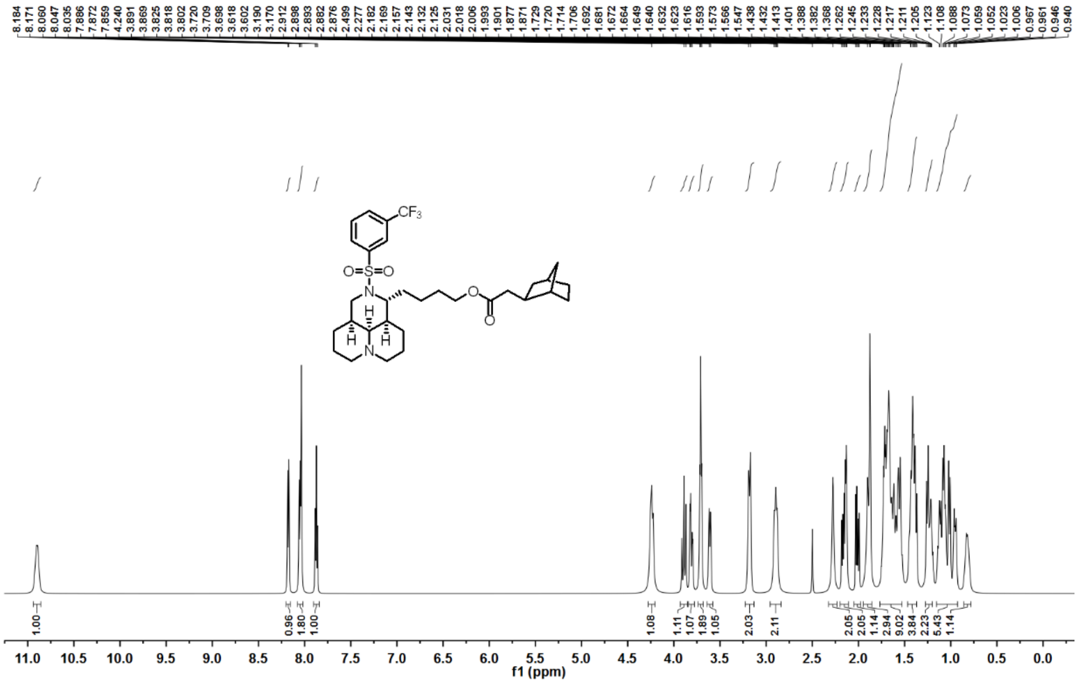

Figure 19S. ^{1}H NMR spectrum of compound of 9j

CARBON_01
VNS-600 CARBON 9j IN DMSO Dec 1 2017

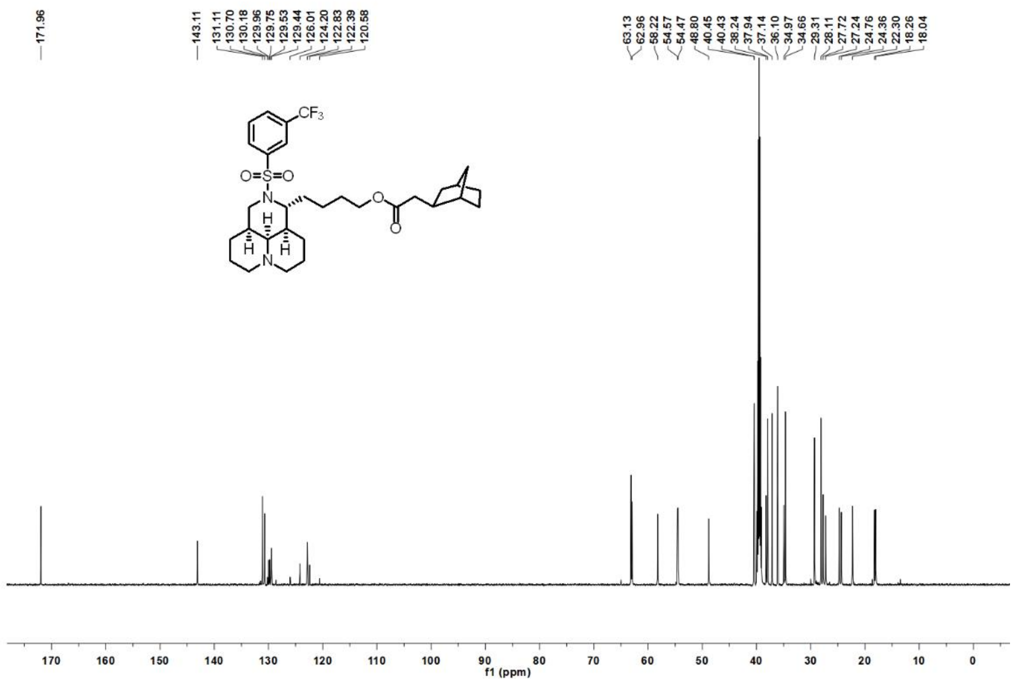

Figure 20S. ^{13}C NMR spectrum of compound of 9j

PROTON_01
VNS-600 PROTON 9k IN DMSO Dec 1 2017

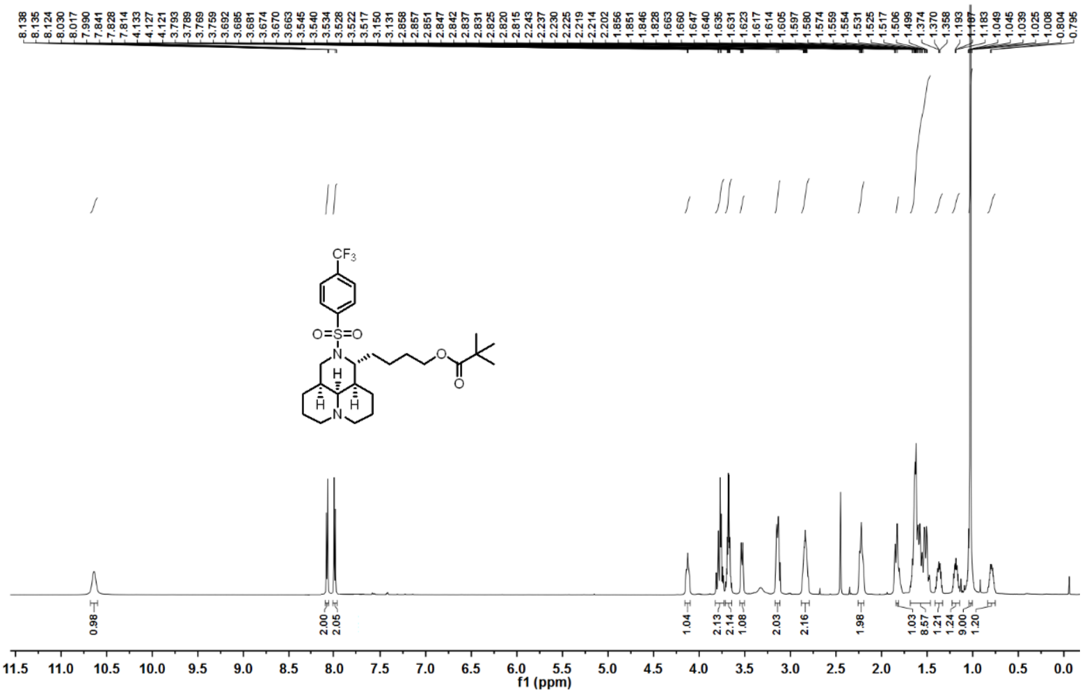

Figure 21S. ^{1}H NMR spectrum of compound of 9k

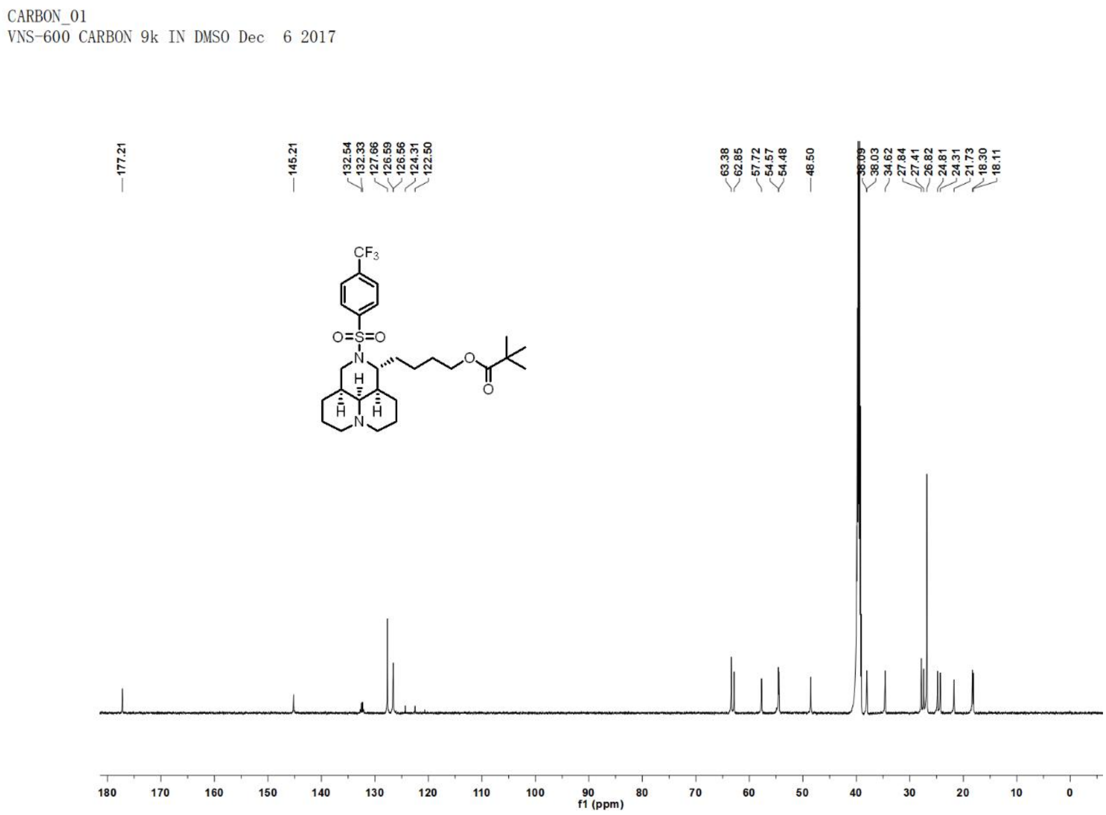

Figure 22S. ^{13}**C NMR spectrum of compound of 9k**

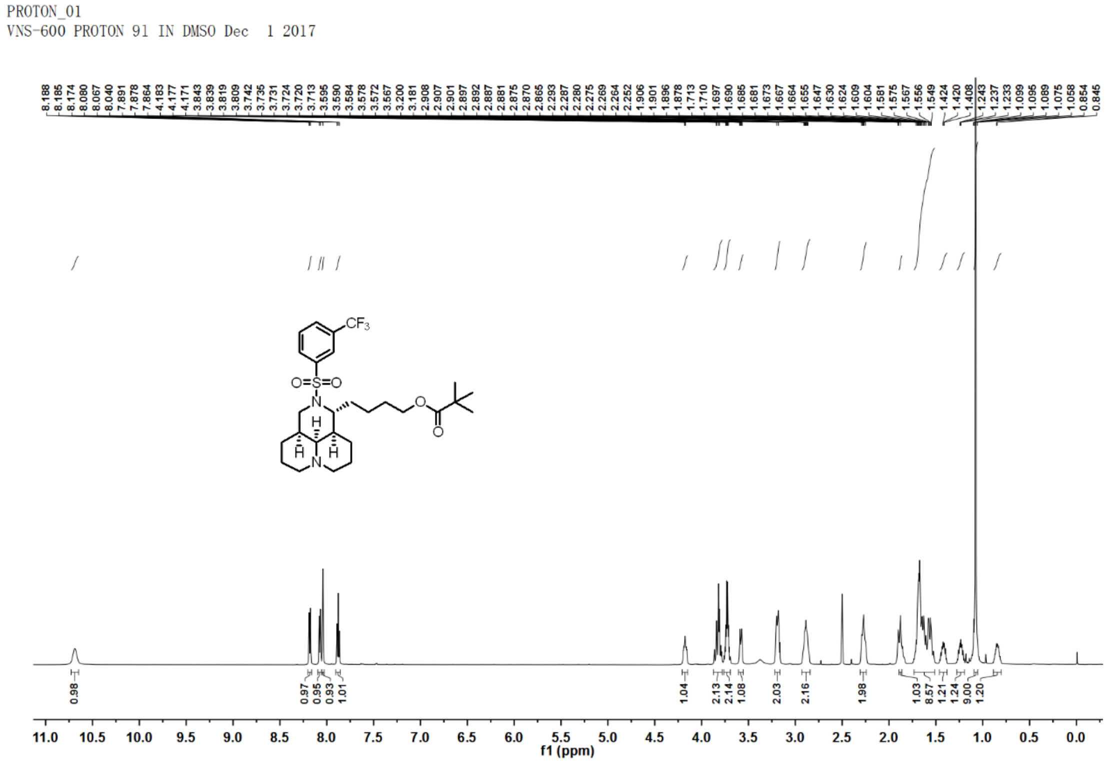

Figure 23S. ^{1}**H NMR spectrum of compound of 9l**

CARBON_01
VNS-600 CARBON 91 IN DMSO Dec 1 2017

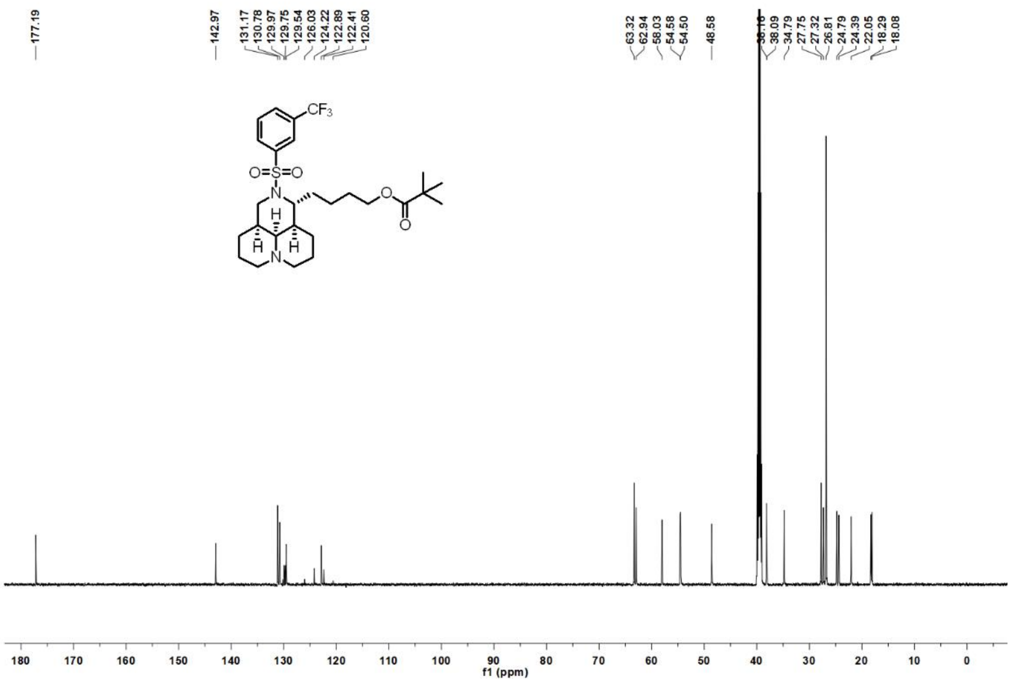

Figure 24S. ^{13}C NMR spectrum of compound of 9l
